# Evidence for balancing selection on loci associated with host use in the genome of a generalist plant parasite

**DOI:** 10.1093/evlett/qrag014

**Published:** 2026-05-07

**Authors:** McCall Calvert, Alison Chen, Adya Gupta, Linda Wu, Corlett Wood

**Affiliations:** Department of Biology, University of Pennsylvania, Philadelphia, United States; Department of Biology, University of Pennsylvania, Philadelphia, United States; Department of Biology, University of Pennsylvania, Philadelphia, United States; Department of Biology, University of Pennsylvania, Philadelphia, United States; Department of Biology, University of Pennsylvania, Philadelphia, United States

**Keywords:** generalist, host–parasite interaction, heterogeneous selection, *Meloidogyne hapla*, root-knot nematode, balancing selection

## Abstract

Most research on host–parasite interactions has focused on tightly coevolved species pairs, yet many parasites are generalists that infect a wide range of host taxa. Generalist parasites are subject to highly variable selection pressures across different hosts and the footprints that this heterogeneous selection leaves in the genomes of generalists have been largely unexplored. To address this knowledge gap, we studied patterns of host-associated genetic diversity in the generalist plant parasite, the northern root-knot nematode *Meloidogyne hapla*. By leveraging whole genome amplification, we generated whole genome libraries of individual root-knot nematodes collected from 3 different host plant species in a spatially replicated design. Despite finding no evidence of host-associated population structure, we did observe several hundred host-associated single nucleotide polymorphisms (SNPs) putatively under differential host selection. Genomic regions containing these host-associated SNPs were enriched for signals of balancing selection. Consistent with balancing selection, polymorphisms originated earlier in genomic windows that contained a host-associated SNP compared to those that did not. Additionally, host-associated SNPs were enriched for functions involved in plant pathogenesis, specifically the modification of plant cell walls. Host-associated SNPs were also found in genes with known roles in dampening host plant immune responses to infection. Together, our results suggest a model of balancing selection in which heterogeneous selection by different host taxa maintains adaptive diversity at loci involved in pathogenesis.

Hosts impose strong selection on parasites. This strong selection maintains high amounts of diversity in pathogen populations, particularly in genetic loci involved in virulence (Ebert & Fields, 2020). The bulk of theoretical and empirical work on host–parasite interactions has focused on tightly coevolved pairs (Clarke, 1976; Ebert, 2008; Hamilton, 1980; Jaenike, 1978). This work has led to well-developed theory and clear predictions on the evolutionary dynamics of coevolved interactions (Dybdahl & Lively, 1998; Lively, 1987; Morran et al., 2011; Papkou et al., 2019; Paterson et al., 2010; Thrall et al., 2012). Yet, many host–parasite interactions lack this tight specificity: hosts are attacked by multiple parasite species and most parasites can invade multiple host species (Barrett et al., 2009; Gibson, 2019; Power & Mitchell, 2004; Woolhouse et al., 2001).

The majority of parasite taxa are generalists (Barrett et al., 2009). The heterogeneous selection pressure exerted by different host species can either favor the evolution of broadly adapted, generalist genotypes or multiple specialist genotypes (Bono et al., 2017; Felsenstein, 1976; Kassen, 2002; Levins, 1968; Visher & Boots, 2020). To date, population genetic studies of generalists have been primarily focused on whether these species are true generalists or are composed of distinct host-associated populations (i.e., host ecotypes) (Cole & Viney, 2018; Montarry et al., 2021). Such studies have historically relied on small marker sets, such as microsatellites or mitochondrial DNA, to do so.

However, even in the genomes of true generalists, some regions may be subject to conflicting host selection pressures. Whenever different alleles are favored on different hosts, such regions should carry signals of balancing selection. The results of experimental evolution studies in the lab that impose heterogeneous selection are consistent with this prediction (Bono et al., 2017; Kassen, 2002). However, the extent of host-associated genetic diversity in wild populations of generalist parasites is still underexplored (but see Vidal et al., 2019). This is largely because parasites are small, challenging to isolate, and difficult to rear in the lab, making individual-level whole genome data from natural populations hard to collect. Furthermore, the small marker sets that have historically been applied to wild generalist parasites are insufficient to detect genomic signatures of selection. Characterizing the genomic signatures of differential selection across host species in a generalist parasite is crucial to understand the evolutionary dynamics of diffuse host–parasite interactions.

In this study, we leveraged whole genome amplification of individual microparasites to interrogate the genomic signatures of selection in a generalist parasite of plants: the northern root-knot nematode (*Meloidogyne hapla*). *Meloidogyne hapla* is diploid, predominately self-fertilizing (but capable of outcrossing), a destructive agricultural pest, and one of the most extreme generalists known to science, with over 80 documented host plant families (Ferris et al., 2024; Jones et al., 2013). *Meloidogyne hapla* invades host plants via root tips and migrates to a desirable feeding site near the primary phloem (Eisenback & Triantaphyllou, 1991). Once a feeding site is found, *M. hapla* penetrate host cells with their stylets and inject effectors that suppress plant immune responses, act as plant hormone mimics, and modify plant cell walls (Castagnone-Sereno et al., 2013; Mitchum & Liu, 2022). These effectors also facilitate the de-differentiation of host cells into enlarged multinucleate “giant cells,” which supply nematodes with their nutrient requirements and lead to the formation of visible root galls (Bali & Gleason, 2024; Eisenback & Triantaphyllou, 1991; Mitchum & Liu, 2022). Genotypes of *M. hapla* show little variation in host species breadth, but can differ substantially in their host-specific performance (Liu & Williamson, 2006). Like other root-knot nematode species, *M. hapla* possesses limited dispersal ability and therefore has little control over the host species they encounter (Prot, 1980). As a result, it is likely that *M. hapla* experiences heterogeneous selection by different host species across space and through time.

In this study, we resequenced the genomes of 78 *M. hapla* individuals collected from root galls on field-sampled plants in a spatially replicated design across four sampling sites. Within each site, we sequenced approximately equal numbers of nematodes from each of three host species (Supplementary Table S1): black medick (*Medicago lupulina*), white clover (*Trifolium repens*), and oxeye daisy (*Leucanthemum vulgare*) (Figure 1A). By comparing the genomes of individuals collected from different host plants, we addressed the following questions: (1) Is there evidence for host-specialized ecotypes? (2) Is there evidence for adaptive genetic variation involved in host adaptation? (3) Are loci putatively involved in differential host adaptation predominantly under positive or balancing selection? And (4) Are loci putatively involved in differential host adaptation enriched for molecular functions involved in plant infection? We also explored patterns of selection on *Meloidogyne* genes previously demonstrated to be involved in plant infection. We found no evidence for host-associated ecotypes. However, *M. hapla* populations still contained extensive host-associated genetic variation. Host-associated loci were enriched for signals of balancing selection and regions under balancing selection tended to be older than the rest of the genome. Finally, host-associated loci were enriched for molecular functions likely involved in the modification of plant cell walls, which is a key process in the formation of their feeding sites within galls. Together, our results suggest that balancing selection driven by heterogeneous selection by different host species maintains variation in the genome of this generalist parasite.

**Figure 1:**
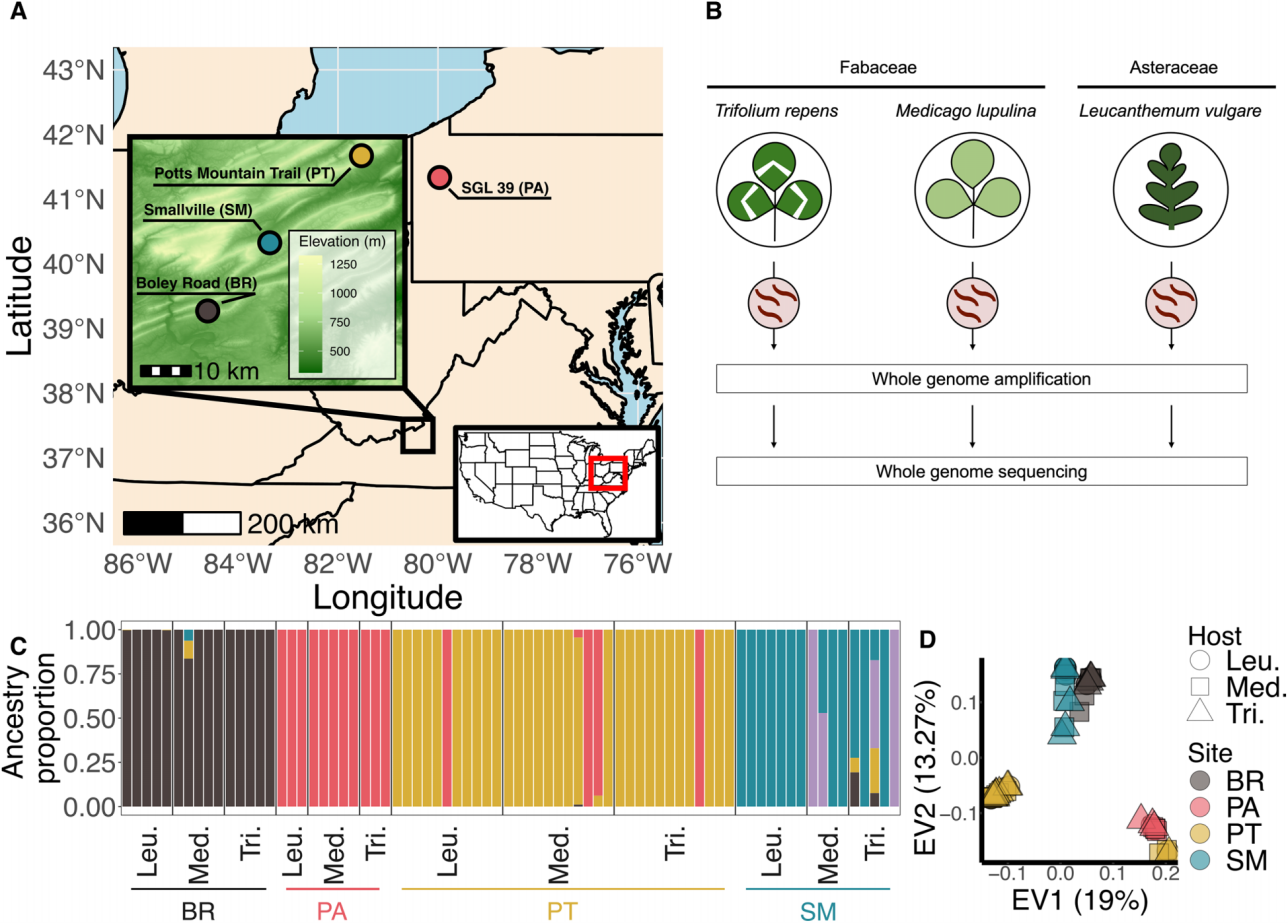
Sampling locations, sampling design, and population structure for the root-knot nematodes collected in this study. (A) Sampling locations in Virginia and Pennsylvania. (B) Sampling design for this study. Root-knot nematode-infected *Trifolium repens, Medicago lupina*, and *Leucanthemum vulgare* were collected at each sampling location. Whole genome amplification followed by whole genome sequencing was performed on individual root-knot nematodes dissected from host plant roots. (C) Ancestry proportions for each individual root-knot nematode calculated using fastSTRUCTURE with *K = 5* (the optimal number of genetic clusters) and colored by the dominant cluster at each sampling location. Each bar represents an individual root-knot nematode and are grouped by the host plant species they were sampled from nested within sampling location. (D) A principal component analysis (PCA) plot showing the first and second eigenvectors of genetic variation in the root-knot nematodes sampled. Points represent individuals and are colored by sampling location as in panel C. Point shapes correspond to the host plant species that individuals were sampled from. In panels C and D, host plant species are abbreviated; Leu. = *Leucanthemum vulgare*, Med. = *Medicago lupulina*, and Tri. = *Trifolium repens*.

## Results

Whole genome libraries for individual nematodes were produced using whole genome amplification (Figure 1B). Raw reads were aligned to the existing chromosome-scale *M. hapla* reference assembly (Shakya et al., 2025), which is 59.2 Mb in size, comprised of 16 chromosomes, and containing 11,229 protein coding genes (6,812 of which have at least one annotated gene ontology [GO] term). After alignment to the existing *M. hapla* reference assembly, two samples with poor alignment to the reference assembly were removed from the dataset. After variant calling, and standard variant filtering (see the *Methods* section), we produced a set of 149,188 biallelic single nucleotide polymorphisms (SNPs) for downstream analysis.

### Geography, but not host species, drives population structure in *M. hapla*

We observed a strong effect of geography, but not host species, on *M. hapla* population structure (Figure 1C and D). A fastSTRUCTURE (Raj et al., 2014) analysis found that the most likely number of population clusters was *K* = 5, nearly matching the number of sampling locations. Almost all individuals within each sampling location showed complete or majority assignment to the dominant population cluster in that location. Two samples from SM were assigned to a unique genetic cluster different from the dominant cluster at that site. Four samples from PT were assigned to the same dominant population cluster as individuals from PA (Figure 1C). Phylogenetic analysis of de novo assembled mitochondrial genomes suggests that the PA samples and the four aforementioned PT individuals may belong to a different cytological type of *M. hapla* (Supplementary Figure S1). One potential explanation for the occurrence of these four individuals from the alternative cytological type at PT is a rare long-distance migration mediated by human activity as infected plant material was collected near roadsides. A principal component analysis (PCA) corroborated the findings from fastSTRUCURE (Figure 1D). To explore whether the large genetic differences between the PA population and the three VA populations could be concealing a signal of host-associated population structure in Virginia, we also performed fastSTRUCTURE and PCA on the 61 individuals collected from sites in Virginia (geography and no effect of host species; Supplementary Figure S2).

A redundancy analysis employed the same linkage disequilibrium (LD)-pruned SNPs used above found that sampling location explained 31.1% of genetic variation while host plant explained 0.2%.

### Genome scans reveal host-associated SNPs

We employed two complementary analyses to identify host-associated SNPs, both using an outlier approach implemented in the genome-environment association analysis tool BayPass (Gautier, 2015; Olazcuaga et al., 2020). In our first analysis, we leveraged all 61 samples across the three sampling sites in Virginia to construct a list of globally host-associated SNPs while controlling for population structure. In our second approach, we used only the 29 samples collected at the most well-sampled site, PT, to construct a list of host-associated SNPs local to that site. Each of these approaches involved making three comparisons between each pair of nematode samples collected from each host species: nematodes collected from *L. vulgare* and *M. lupulina* (Leu. vs. Med.), nematodes collected from *L. vulgare* and *T. repens* (Leu. vs. Tri.), and nematodes collected from *T. repens* and *M. lupulina* (Tri. vs. Med.). The *p*-values for SNP *C_2_* statistics estimated by BayPass were non-uniform, and we therefore classified SNPs as host-associated if their standardized *C_2_* was more extreme than the 99th quantile of a pseudo-observed dataset (POD) (Olazcuaga et al., 2020; Supplementary Methods). A permutation test with 1,000 iterations of shuffling the sample labels on the SNP matrix resulted in a coarse null distribution with likely a high number of false positives (Supplementary Methods, Supplementary Figure S3). To limit type I error, we used classifications of host-associated SNPs based on the POD threshold.

**Figure 2:**
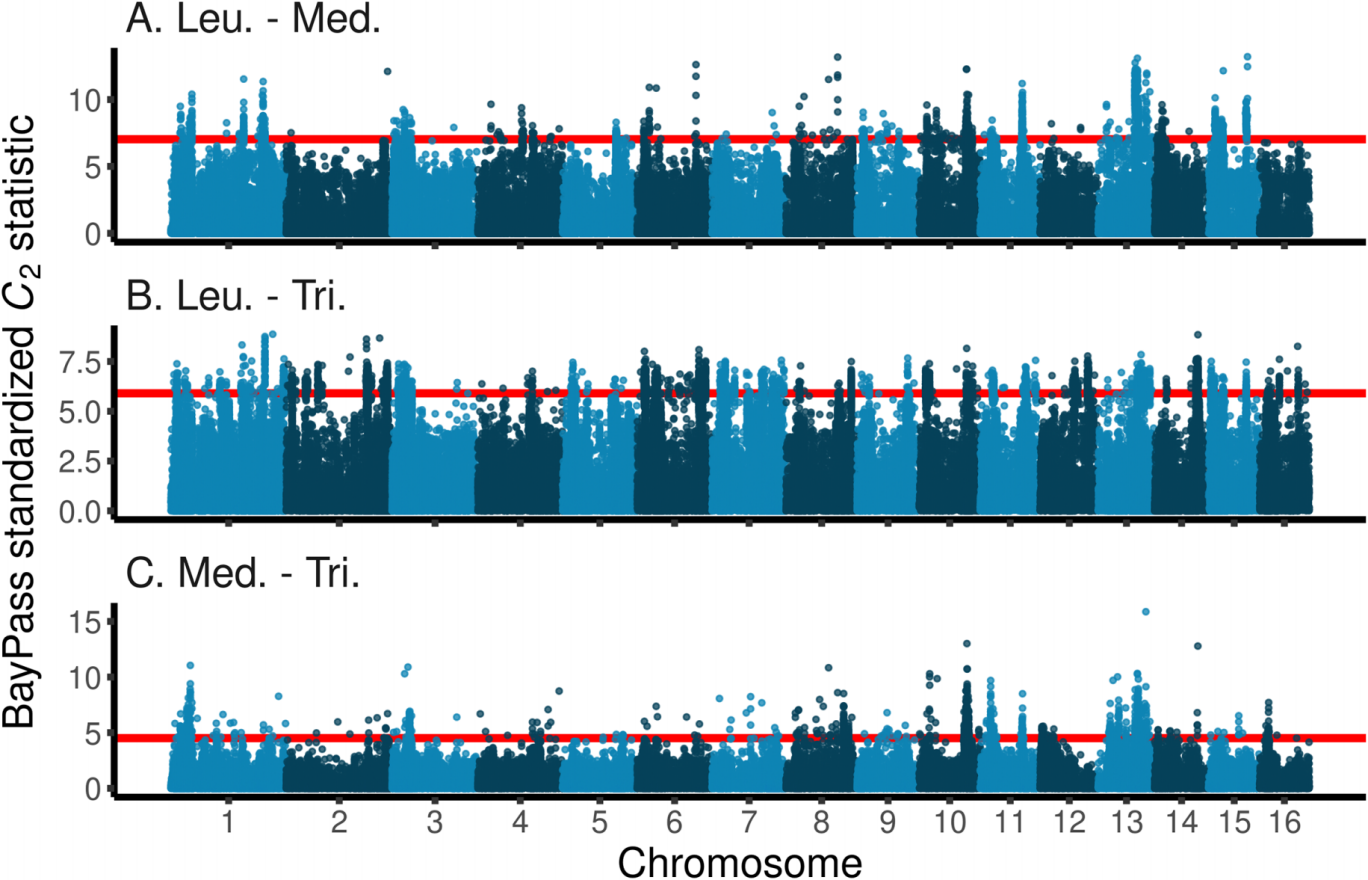
Manhattan plots of BayPass standardized contrast statistics (*C_2_*) generated by comparisons of nematodes collected from different host plants: (A) comparison of nematodes collected from *Leucanthemum vulgare* versus *Medicago lupulina* (Leu.—Med.), (B) comparison of nematodes collected from *Leucanthemum vulgare* versus *Trifolium repens* (Leu.—Tri.), and (C) comparison of nematodes collected Medicago *lupulina* versus *Trifolium repens* (Tri.—Med.). Points represent the *C_2_* of individual SNPs. The horizontal line in each panel represents the 0.99% quantile of the pseudo-observed dataset generated for each specific comparison. Points above the horizontal line are classified as significantly host-associated.

When leveraging samples from all three sampling sites, we found a global set of 4,701 host-associated SNPs: 1,130 in the Leu. vs. Med. comparison, 2,834 in the Leu. vs. Tri. comparison, and 1,030 in the Med. vs. Tri. comparison (Figure 2). Employing BayPass locally at PT revealed a total of 235 locally host-associated SNPs; 43 in the Leu. vs. Med. comparison, 127 in the Leu. vs. Tri. comparison, and 72 in the Med. vs. Tri. comparison (Supplementary Figure S4). Furthermore, population structure-corrected standardized allele frequency differences were strongly correlated between the global and local analyses (Supplementary Figure S5).

### Host-associated SNPs are enriched for balancing selection

To determine whether any host-associated SNPs are putatively under positive or balancing selection, we quantified Tajima’s *D* in 10-kb, non-overlapping windows in our most well-sampled site, PT (see the *Methods* section). Positive values of Tajima’s *D* indicate an excess of intermediate-frequency polymorphisms, consistent with balancing selection; negative values of Tajima’s *D* indicate an excess of low-frequency polymorphisms, consistent with a recent selective sweep (i.e., positive selection). Positive and negative Tajima’s *D* values can also be indicative of recent population expansions or contractions, respectively (Tajima, 1989). Our demographic inference using coalescent simulations in fastsimcoal found very little population growth (Supplementary Table S2), suggesting population size changes have a minimal effect on observed Tajima’s *D* values.

**Figure 3:**
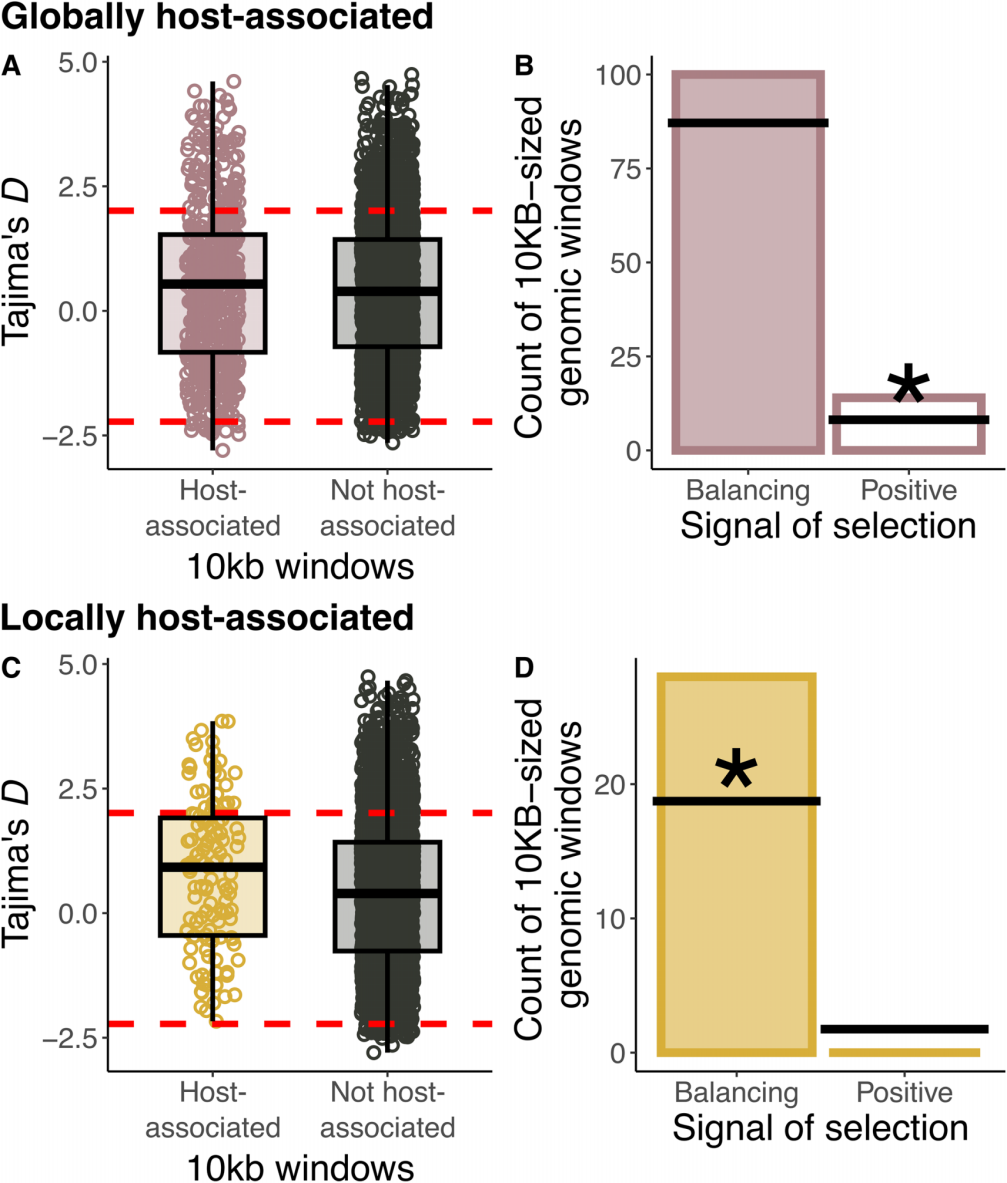
Signals of selection in genomic windows harboring (A, B) globally host-associated SNPs and (C, D) locally host-associated SNPs compared to the rest of the genome. Distributions of Tajima’s *D* values are plotted for 10-kb windows that do versus do not contain (A) globally host-associated SNPs and (C) locally host-associated SNPs. In these panels, each point represents Tajima’s *D* value for a single 10-kb window. Dashed horizontal lines represented the empirical 0.5% and 99.5% significance thresholds for determining the significance of a signal for selection using simulated genetic data. Tests for enrichment of significantly positive (balancing selection) and significantly negative (positive selection) Tajima’s *D* values in genomic windows that contain globally host-associated SNPs and locally host-associated SNPs are shown in panels B and D, respectively. Panels B and D contain bar plots of the observed number of genomic windows that contain host-associated SNPs and exhibit a significant signal of balancing (shaded bar) or positive selection (open bar). Solid horizontal lines mark the expected number of genomic windows in a given category and stars indicate significant enrichment tested using a Fisher exact test.

We found that 10-kb windows containing host-associated SNPs had higher Tajima’s *D* values than the rest of the genome, slightly so for 10-kb windows with globally host-associated SNPs (*F*_1,2946_ = 0.6312, *p* = 0.427), and significantly so for 10-kb windows with host-associated SNPs identified locally at PT (*F*_1,2905_ = 7.5385, *p* = 0.006) (Figure 3A and C).

We then tested for enrichment of host-associated SNPs in regions that exhibited signals of selection. To threshold for significant signals of selection, we used the 0.5% and 99.5% quantiles of a distribution of Tajima’s *D* values calculated from genetic data simulated from a best-fitting demographic model (see the *Methods* section). Ten-kb windows exhibiting significantly positive values of Tajima’s *D* were more common than those with significantly negative values. However, only 10-kb windows containing host-associated SNPs identified locally at PT were significantly enriched for significant positive values of Tajima’s *D* (odds ratio = 1.68, *p* = 0.02; Figure 3B and D).

Many host-associated SNPs are likely to be at an intermediate allele frequency in the PT population as a whole. Therefore, enrichment of positive Tajima’s *D* values could result from alleles at these loci existing at an intermediate frequency, rather than sustained balancing selection. When matching genomic windows by the number of SNPs they contained and their mean allele frequency, genomic windows containing host-associated SNPs identified locally at PT still had significantly higher Tajima’s *D* values (Wilcoxon signed rank test, *p* = 0.005; Supplementary Figure S6), but genomic windows containing host-associated SNPs identified globally across the entire dataset did not (Wilcoxon signed rank test, *p* = 0.247; Supplementary Figure S6).

### Genomic windows with signals of balancing selection are older than putatively neutral regions

Balancing selection is expected to maintain selected alleles at intermediate frequencies for extended periods of time. Therefore, regions under balancing selection should be older than regions under positive selection. Tests that compare the age of genetic variation in windows with significantly positive Tajima’s *D* values versus the rest of the genome are useful in determining whether a significantly positive Tajima’s *D* is a result of balancing selection or another evolutionary process that can inflate Tajima’s *D* values, such as recent changes in population size or population structure (Nordborg & Innan, 2003; Takahata, 1990). We estimated the time to the most recent common ancestor (TMRCA) for SNPs averaged across the same 10-kb windows used in the Tajima’s *D* analysis using ARGweaver (Rasmussen et al., 2014). ARGweaver estimates ancestral recombination graphs (ARGs), which are complete records of all recombination and coalescence events since divergence among sequences in a population sample. As such, ARGs represent a complete genealogy of every genomic position under study. If a given locus is under balancing selection, then its TMRCA should be longer than a locus under weak selective constraints (i.e., neutral).

**Figure 4:**
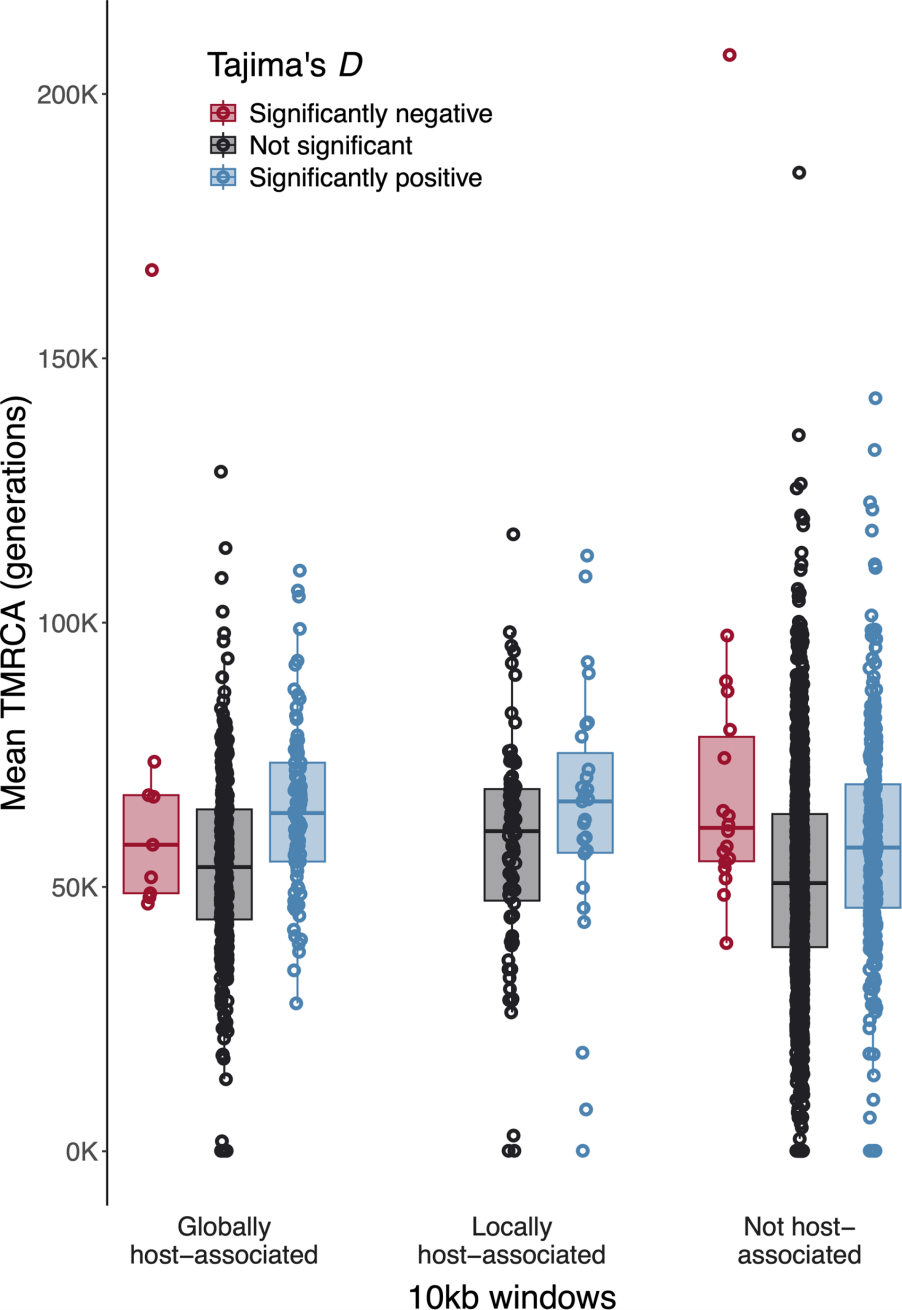
Estimates of time to the most recent common ancestor (TMRCA) of genomic positions containing SNPs. Each point represents the mean TMRCA for all positions within a 10-kb window.

We found that windows with significantly positive Tajima’s *D* values contained polymorphisms that were, on average, significantly older than polymorphisms in windows with significantly negative Tajima’s *D* values and windows with non-significant Tajima’s *D* values (*F*_1,2570_ = 37.52, *p* < 0.00019; Figure 4). Similarly, host-associated SNPs were, on average, significantly older than windows that did not contain host-associated SNPs (*F*_1,2570_ = 10.3600, *p* < 0.0001). Among windows containing host-associated SNPs identified using samples from all sites (globally host-associated), the mean TMRCA of windows with significantly positive Tajima’s *D* values were significantly greater than either windows with significant negative Tajima’s *D* or non-significant Tajima’s *D* values (*F*_1,486_ = 16.211, *p* < 0.0001). Meanwhile, among windows containing host-associated SNPs identified locally at the PT site, the mean TMRCA of windows with significantly positive Tajima’s *D* were, on average, older than windows that did not contain host-associated SNPs, but the trend was not significant (*F*_1,112_ = 1.26, *p* = 0.264).

### Host-associated SNPs are enriched for functions putatively involved in plant cell wall modification and enriched in putative regulatory regions

We performed tests for enrichment on GO terms on cellular component (CC), molecular function (MF), and biological process (BP) separately on global host-associated SNPs and local PT host-associated SNPs. Global host-associated SNPs were enriched in many CC GO terms linked to the cell surface and extracellular space, specifically cell periphery (GO:0071944), plasma membrane (GO:0005886), cell cortex region (GO:0099738), membrane (GO:0016020), and basement membrane (GO:0005604), suggesting a potential role in effector secretion and host cell recognition (Table 1). We also observed enrichment of global host-associated SNPs in some MF GO terms with potential roles in plant cell wall modification or degradation, specifically hexosaminidase activity (GO:0015929), β-*N*-acetylhexosaminidase activity (GO:0004563), phosphatidylinositol phosphate phosphatase activity (GO:0052866), and phosphatidylinositol bisphosphate phosphatase activity (GO:0034593) (Supplementary Table S3). In contrast, global host-associated SNPs were enriched in BP GO terms linked to homeostasis, sugar metabolism, and development (Supplementary Table S4).

**Table 1:** Significantly enriched cellular component GO terms for global host-associated SNPs.

GO.ID	Term	Annotated	Significant	Expected	*p*-value
GO:0071944	Cell periphery	582	55	40.66	0.0088
GO:0016272	Prefoldin complex	7	3	0.49	0.0096
GO:0061908	Phagophore	3	2	0.21	0.0139
GO:0005886	Plasma membrane	538	50	37.58	0.0172
GO:0099738	Cell cortex region	4	2	0.28	0.0266
GO:0016020	Membrane	1,320	107	92.21	0.0288
GO:0005604	Basement membrane	5	2	0.35	0.0422
GO:0005881	Cytoplasmic microtubule	5	2	0.35	0.0422
GO:0034388	Pwp2p-containing subcomplex of 90S preribosome	5	2	0.35	0.0422

Many of the enrichment tests for host-associated SNPs identified locally at the PT site were also underpowered (Supplementary Tables S5–S7). However, of particular note was the significant enrichment of CC GO terms extracellular region (GO:0005576) and extracellular space (GO:0005615), which, similar to patterns of GO term enrichment in global host-associated SNPs, suggests roles in effector secretion or host cell recognition.

We also performed CC, MF, and BP GO term enrichment tests on global host-associated SNPs and local PT host-associated SNPs located in genomic windows with significantly positive Tajima’s *D* values (signal of balancing selection). Similar to the results presented above, enrichment for CC, MF, and BP in both global and local host-associated SNPs residing in genomic windows with significantly positive Tajima’s *D* values produced significant GO terms that pointed to functions in effector secretion, plant cell wall modification, and development, but were largely underpowered (Supplementary Tables S8–S13). No host-associated SNPs in genomic windows with significantly negative Tajima’s *D* values (signal of positive selection) were located in genes with annotated GO terms.

**Figure 5:**
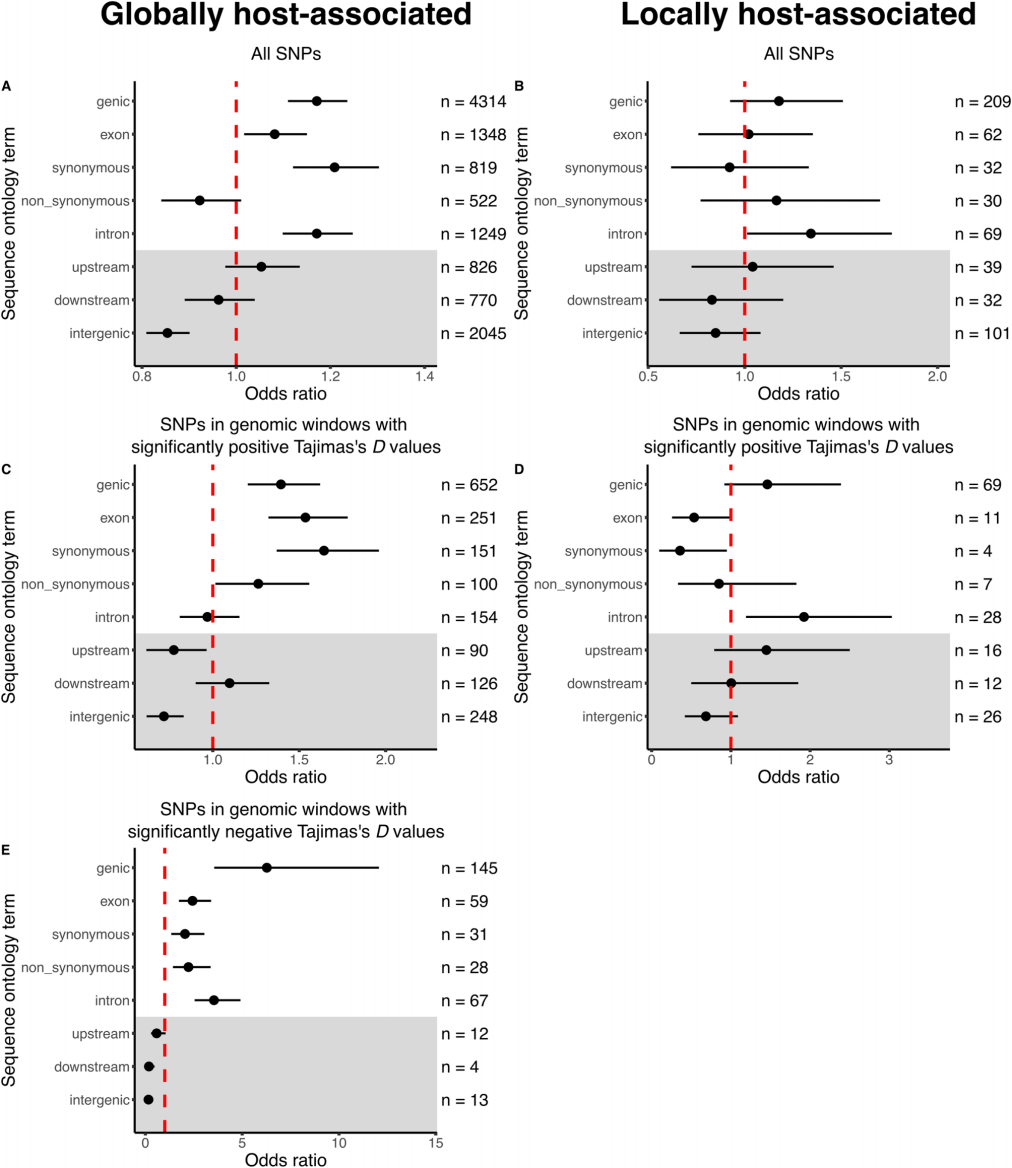
Sequence ontology (SO) enrichment analysis for (A, C, E) global SNPs and (B, D) local PT SNPs. Panels A and B display results of SO enrichment tests on all SNPs from each sample set, panels C and D display results of SO enrichment tests on SNPs residing in 10-kb windows with significantly positive Tajima’s *D* values, and panel E displays the results of SO enrichment tests on SNPs residing in 10-kb windows with significantly negative Tajima’s *D* values. No local PT SNPs were contained in windows with significantly negative Tajima’s D values. Points are the odds ratios derived from Fisher tests and bars are the 95% confidence intervals around the odds ratio. SO terms with error bars that do not overlap with one (the dashed line) are significantly enriched. SO terms with odds ratios below one (to the left of the dashed line) are underenriched, while SO terms with odds ratios above one (to the right of the dashed line) are enriched. The number of significant SNPs that fall into a given SO term is listed to the right of each plot.

Furthermore, a sequence ontology analysis revealed that global host-associated SNPs were significantly enriched in genic regions, a pattern that appears to be driven by enrichment of SNPs in introns, and synonymous changes in exons (Figure 5A). Similar enrichment of introns was also observed in locally host-associated SNPs (Figure 5B). In contrast, significant enrichment in genic regions of global host-associated SNPs, with significantly positive Tajima’s *D* values, appears to be driven by enrichment of SNPs in exons, and both synonymous and nonsynonymous changes (Figure 5C). Locally host-associated SNPs with significantly positive Tajima’s *D* values were enriched only in introns (Figure 5D). Lastly, globally host-associated SNPs with significantly negative Tajima’s *D* values were enriched in genic regions, exons, nonsynonymous changes, synonymous changes, and introns (Figure 5E).

### Evidence for selection on genes and loci with previously identified roles in plant infection

We also searched for host-associated SNPs in a list of 191 effectors and plant cell wall-modifying genes that have a known or suspected role in plant infection in *Meloidogyne* (Supplementary Table S14). Global host-associated SNPs were found in 24 of these genes (Supplementary Table S14), more than was expected by chance (odds ratio = 1.58, *p*-value = 0.047)

**Figure 6:**
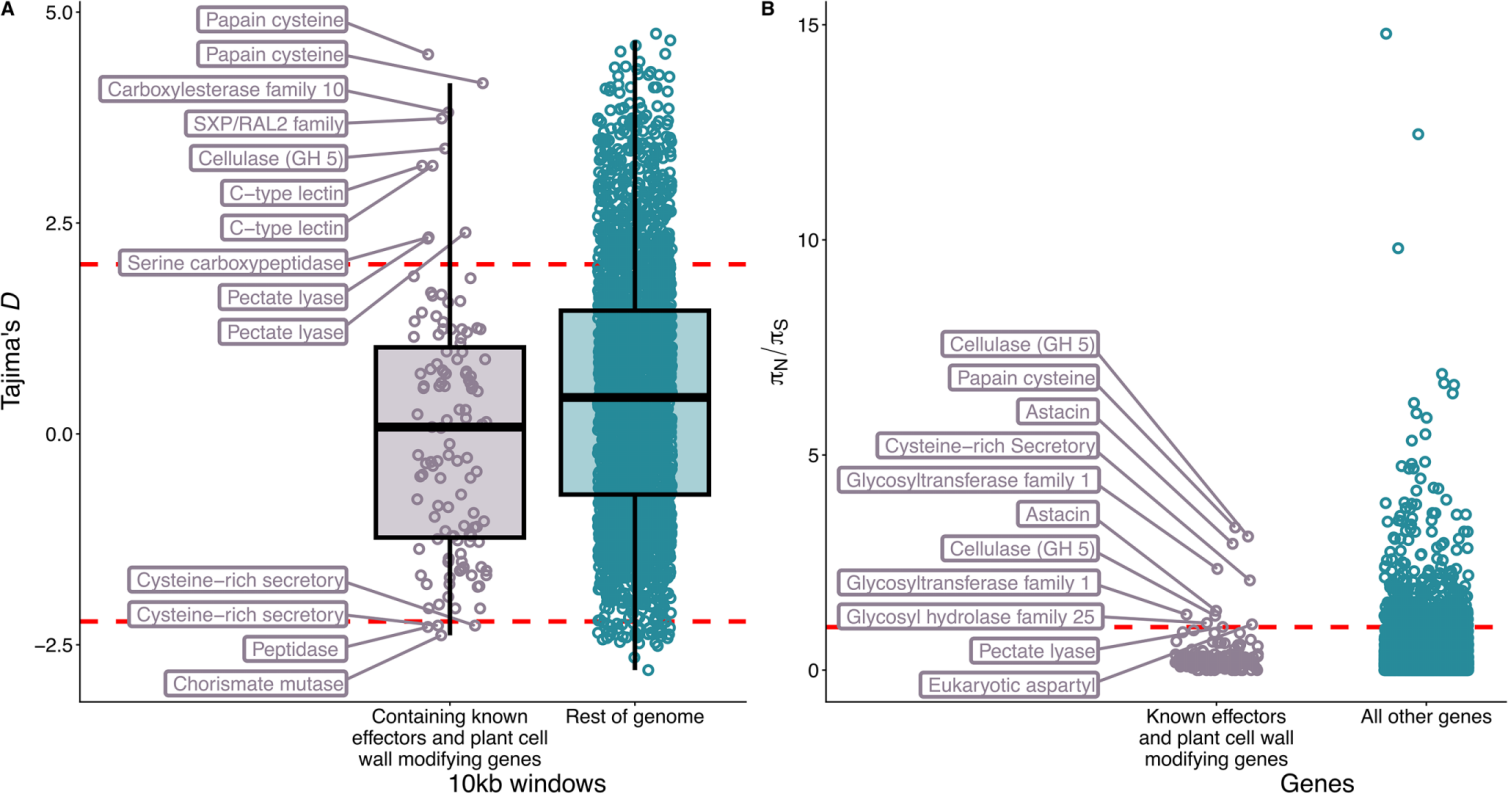
Signals of selection in loci with known roles in plant infection. (A) Comparison of the distribution of Tajima’s *D* values for 10-kb genomic windows that contain known effectors or plant cell wall-degrading enzymes versus the rest of the genome. Points represent genomic windows containing annotated genes. Genomic windows with significantly positive or negative Tajima’s *D* values that contain known effectors and plant cell wall-modifying genes are labeled. Note that the Pectate lyase gene (*ACQ4LE_008949*) crosses a 10-kb window boundary and so appears twice. The dashed lines represent the significance thresholds for Tajima’s *D* as determined via genetic simulations from the best-fitting demographic model (see the *Methods* section) (B) Comparison of π_N_/π_S_ estimates for known effectors and plant cell wall-degrading enzymes compared to the rest of the genome. Points represent the average π_N_/π_S_ value for each gene. Known effectors and plant cell wall-modifying genes with π_N_/π_S_ > 1 (above the dashed line) are labeled.

Fourteen of the known effectors and plant cell wall-modifying genes were located in 10-kb windows with signals of selection: 10 in windows with signals of balancing selection and 4 in windows with signals of positive selection (Figure 6A). Of particular note was a cellulase gene (*ACQ4LE_007320*) and a pectate lyase gene (*ACQ4LE_008949*) residing in genomic windows with signals of balancing selection. These genes are likely involved in degradation and reorganization of plant cell walls. However, as a group, the genomic windows containing the 191 known effectors and plant cell wall-modifying genes had Tajima’s *D* values that were significantly lower than from the rest of the genome (*F*_1,2962_ = 9.2825, *p* = 0.0023; Figure 6A).

Finally, we examined signals of selection on the coding sequences of these 191 genes using π_N_/π_S_ (Hughes & Nei, 1988; Nei & Gojobori, 1986). One hundred fifty of the 191 known effectors and plant cell wall-modifying genes harbored polymorphism in coding sequences with most exhibiting π_N_/π_S_ estimates < 1, indicative of purifying selection (Figure 6B). Eleven genes had π_N_/π_S_ > 1, which could either be a signal of strong balancing selection maintaining multiple alleles or weak purifying selection. The known effectors and plant cell-modifying genes were not more likely to have π_N_/π_S_ > 1 relative to the rest of the genome (odds ratio = 1.3439, *p* = 0.3656), but their mean π_N_/π_S_ of 0.356 was significantly larger than the rest of the genome mean π_N_/π_S_ of 0.317 (χ^2^ = 4.06, *p* = 0.0439).

## Discussion

We conducted a population-based resequencing study of root-knot nematode parasites collected from three different host species at multiple geographic sites. Despite no evidence for host-specialized ecotypes, we did find hundreds of loci that appear to be under divergent selection by different host plant species. Many of these SNPs have likely roles in plant infection, including the modification of plant cell walls—a key process in the formation of the root galls that serve as nematode feeding sites (Bali & Gleason, 2024; Mitchum & Liu, 2022). We also found that a large number of host-associated SNPs are the targets of balancing selection and that regions under balancing selection contained polymorphisms that, on average, originated earlier than polymorphisms in the rest of the genome. Furthermore, we found several previously identified effectors appear to also be targets of balancing selection imposed by different hosts. Our results suggest that different host species select for and maintain adaptive diversity in this generalist parasite. This hypothesis represents an important alternative hypothesis to the prevailing coevolutionary framework in parasite evolutionary genomics: that balancing selection in parasite genomes is indicative of ongoing Red Queen coevolution with a single host. Our study suggests that selection imposed by entire host communities, rather than single taxa, may shape adaptive variation in parasites.

### Host use drives allele frequency differentiation, but not the formation of host-associated ecotypes

Our population structure analyses show that *M. hapla* is a true generalist, rather than a collection of host-specialized ecotypes (Figure 1). This is consistent with what is known about *M. hapla* life history: root-knot nematodes, including *M. hapla*, are weak dispersers, and thus have limited control over which hosts are available for them to infect (Pinkerton et al., 1987; Prot, 1980). Such conditions are predicted to favor the evolution of true generalists (Felsenstein, 1976; Kassen, 2002; Levins, 1968). Our finding that *M. hapla* is a true generalist may also explain its status as a common and destructive agricultural pest. Spillovers and cross-species transmissions are expected to be more frequent in true generalists (Power & Mitchell, 2004; Woolhouse et al., 2001).

Despite *M. hapla*’s identity as a true generalist, we found several hundred SNPs with differentiated allele frequencies between nematodes collected from different host plants indicating that *M. hapla* populations do respond to differential selection by hosts (Figure 2). Contrary to our initial expectation, the comparison between nematodes isolated from the most closely related host pair—*M. lupulina* and *T. repens*—produced some of the most extreme *C_2_* values (a measure of allele frequency differentiation that accounts for population structure). This finding suggests that, at least in some cases, the response to differential host selection is independent of host phylogeny. Weak phylogenetic signal in parasite adaptation to different hosts is possible if phylogenetically distant hosts share traits that produce similar selective pressures (de Vienne et al., 2013; Futuyma & Moreno, 1988). For example, both *T. repens* and *L. vulgare* have shallow and non-woody root systems relative to *M. lupulina* (Chmelíková & Hejcman, 2012). Root physiology is likely important in *M. hapla* infection success (Eisenback & Triantaphyllou, 1991).

Consistent with root physiology shaping selection, we found that globally host-associated SNPs were enriched for genes with GO terms with likely functions in plant cell wall modification and host cell recognition as well as in a list of known effector and plant cell wall-modifying enzymes. Specifically, globally host-associated SNPs were overrepresented in genes involved with hexosaminidase and β-*N*-acetylhexosaminidase activity. Hexosaminidases are responsible for cleaving terminal GlcNAc or GalNAc residues from sugars (Ghosh et al., 2011 ; Strasser et al., 2007). Plant cell walls are studded with key structural and defensive proteins that often contain GlcNAc-modified glycans (Nguema-Ona et al., 2014). Thus, these enzymes may be involved in cleaving protective host cell wall proteins and loosening structural glycoproteins. We also found host-associated SNPs in several genes known to be involved in plant cell wall modification. These included two pectate lyases (*ACQ4LE_008379* and *ACQ4LE_008373*), which are responsible for cleaving pectin, a key component of plant cell walls; a cellulase (*ACQ4LE_007,056*), which is responsible cleaving cellulose, another key structural component of plant cell walls; and two astacins (*ACQ4LE_006401* and *ACQ4LE_000297*), which remodel extracellular space and facilitate tissue migration (Chen et al., 2021; Danchin et al., 2010; Lilley et al., 2018; Stepek et al., 2015). We hypothesize that these genes in *M. hapla* facilitate the modification of plant cells into the giant cells that serve as the root-knot nematode feeding site. Additionally, the observation of host-associated SNPs in genes that manipulate the plant immune response suggests that plant cell wall modification is likely not the sole target of selection (Supplementary Table S14). Lastly, the enrichment of global host-associated SNPs in several cellular component GO terms linked to cell membranes and extracellular space suggests that many host-associated SNPs are involved in effector secretion and host plant cell recognition (Table 1).

Further emphasizing a functional role of the host-associated SNPs we uncovered was their overrepresentation in genic regions, including in both exons and introns. Global host-associated SNPs were enriched in exons, which suggests a potential role for variation in amino acid substitution in facilitating responses to selection by alternative host plants (Figure 5A). Accordingly, nonsynonymous changes in exons were enriched in genomic regions with significant signals of balancing selection (Figure 5C and E). Host-associated SNPs in both the global and local datasets were enriched in introns, which implicates a potential role for variation in the expression of specific gene isoforms, via changes to splice sites or splicing regulatory sequences, underpinning the response to differential host selection (Figure 5A and B). To summarize, we found hundreds of SNPs that are putative targets of differential host selection in this generalist parasite. This finding complements and extends theory on the evolution of generalist parasites in two ways. First, the maintenance of polymorphisms in a true generalist is consistent with theory and previous studies on evolution in heterogeneous environments (Felsenstein, 1976; Kassen, 2002; Levins, 1968). Second, the apparent variation in selection exerted by different host species highlights the important role that host community composition plays in the evolution of generalist parasites (Power & Mitchell, 2004; Thompson, 2005; Woolhouse et al., 2001). Our finding of weak genetic differentiation between nematodes isolated from distantly related host plants (*L. vulgare* and *T. repens*) raises the possibility that the phylogenetic structure of host communities may be less important in the evolution of generalists than specialized parasites (Parker et al., 2015).

### Long-term balancing selection maintained by heterogeneous selection across host species

One of the most striking patterns in our dataset is the enrichment of host-associated loci in genomic windows exhibiting a signal of balancing selection as measured by Tajima’s *D* (Figure 3). A similar trend also exists when comparing the average Tajima’s *D* of windows containing host-associated SNPs versus those that do not. Our confidence that these represent signals of balancing deriving from differences in selection imposed by different hosts is strengthened by our observations that polymorphisms in these regions tend to be, on average, older than polymorphisms rest of the genome, as measured by a TMRCA analysis (Figure 4). The prevalence of older polymorphisms in genomic regions with significantly positive Tajima’s *D* estimates supports the hypothesis that balancing selection, rather than another evolutionary process, is maintaining alleles at intermediate frequencies in those regions. Together, our data suggest that heterogenous host selective pressures maintain balanced polymorphisms at loci involved in host infection.

An alternative explanation for the long-term maintenance of host-associated polymorphisms is specific coevolution within one or several different host species (Thompson, 1994). However, given the high variability in which root-knot nematodes encounter different host species, it is unlikely that they infect any specific host species consistently enough to allow coevolution to occur (Gibson, 2019; Gomulkiewicz et al., 2000).

We also explored signals of selection in 191 genes known or suspected to be involved in plant infection (Figure 6; Supplementary Table S14), regardless of whether or not they contained host-associated SNPs. Fourteen known effectors and plant cell wall-modifying genes exhibited signals of selection, 10 resided in genomic windows with signals of balancing selection and 4 resided in genomic windows within signals of positive selection. Of particular note are a pectate lyase (*ACQ4LE_008949*) and a cellulase (*ACQ4LE_007320*) seemingly under balancing selection and a Chorismate mutase gene (*ACQ4LE_003725*), which has a role in suppressing salicylic acid synthesis, under positive selection (Danchin et al., 2010; Feng et al., 2024). Finally, our data suggest that the coding sequences of known effectors are subject to strong selective constraint. Of the 150 known effectors and plant cell wall-modifying genes with polymorphisms in our dataset, 139 had π_N_/π_S_ < 1, consistent with purifying selection. The exceptions included a pectate lyase, a cellulase, and astacins (Figure 6B). Generally π_N_/π_S_ > 1 are interpreted as either weak purifying selection, from, for example, selective interference, or as signals of multiallelic balancing selection, as the number of nonsynonymous mutations that need to be simultaneously segregating to detect π_N_/π_S_ values above one is inconsistent with positive selection (Hahn, 2019). Several immune-related genes in other systems, such as the major histocompatibility complex genes, also contain high values of π_N_/π_S_ presumably because diffuse interactions with several parasite taxa work to maintain many distinct alleles of these genes (Hughes & Nei, 1988; Osborne et al., 2017; Radwan et al., 2020; Spurgin & Richardson, 2010).

Many of the genes analyzed here exhibiting significant signals of selection do not contain host-associated SNPs. This raises the question of what selective agent could be responsible for these observed patterns of nucleotide diversity. One explanation is that these signals of selection are a result of selection by other host plants occurring at the sampling sites where we collected infected root material. While *M. lupina, T. repens*, and *L. vulgare* were common, and sometimes dominant members of the plant communities we sampled, we commonly observed other confirmed *M. hapla* hosts at these sites, such as *Daucus carota, Trifolium pratense*, and *Plantago major*. An exciting avenue for future research is to expand investigations of *M. hapla* to other known hosts to further characterize the role of heterogenous selection by hosts in shaping *M. hapla* diversity.

### Evolutionary genomics of wild parasites

The whole genome amplification approach we employed here gave us the opportunity to explore genome-wide patterns of variation, which revealed extensive evidence of host-driven selection. This method could also be applied in a laboratory setting to quantify the fitness effects of alternative alleles on different hosts to interrogate putative genetic tradeoffs identified in population genomic studies of selection like ours. Whole genome amplification could be an invaluable tool to study the evolutionary dynamics of wild parasites (Cruaud et al., 2018; O’Grady et al., 2022; Pilling et al., 2023; Shortt et al., 2017).

We present evidence that infection-associated SNPs are maintained by balancing selection resulting from diffuse interactions with different host species. The idea that diffuse interactions among multiple parasites or hosts can support the long-term maintenance of polymorphisms is not new, but has been largely explored from the perspective of hosts interacting with several parasite species (Karasov et al., 2014; Radwan et al., 2020). Given that the majority of parasite species are capable of infecting more than one host species (Barrett et al., 2009), the maintenance of polymorphism via diffuse selection from different host species is probably not unique to *M. hapla*. Therefore, future research should be careful when ascribing balancing selection in generalist parasites to pairwise host–parasite coevolution.

Parasites are small and can be difficult to isolate, which has led to a lack of whole genome studies, at least relative to host species. This difficulty also contributed to the relatively small sample sizes that we employed in this study (78 nematodes in total, and 61 for the majority of the inferences on selection). Small sample sizes in population genomics studies result in important constraints, such as limited ability to detect phenotype-associated genetic variation and increased sensitivity of population genetic statistics to demography. With these constraints in mind, we want to emphasize that the main hypothesis that emerges from this study, that host plant diversity begets parasite genetic diversity, should continue to be explored in *M. hapla* and in other generalist parasites.

## Methods

### Sample collection, whole genome amplification, and sequencing

Infected plants belonging to three host plant species (*Medicago lupulina, Trifolium repens*, and *Leucanthemum vulgare*) were collected at each of four study locations, three in Virginia (Boley Field Road [BR], Smallville Fire Access Road [SM], and Pott’s Mountain Trail [PT]), and one in Pennsylvania (State Game Lands 39 [PA]) (Figure 1A). These three host plants were chosen because they were common in both regions of VA and PA where we sampled nematodes. Infected root material was collected by digging up root systems of the three host plant species and examining those root systems for galls. Sampling proceeded at each site until approximately 5–10 infected individuals of each host plant species were collected. Within each site, approximately equal numbers of nematodes from each host plant were selected for sequencing (Supplementary Table S1). After field collections, galls were cut from roots and stored at −80 °C.

Whole adult female nematodes were dissected from frozen gall samples and again stored at −80 °C. Only one nematode per infected root sample was selected for sequencing. Next, we performed whole genome amplification on individual whole nematodes (see Supplementary Methods for full protocol). It should be noted that whole genomic amplification caries the opportunity for PCR bias that may deflate the frequency of heterozygous genotype calls. A thorough examination of PCR bias in whole genome amplification by Lack et al. (2018) found that the effect of PCR bias on heterozygous genotype calls was negligible. We confirmed species identity of amplified samples with PCR using the diagnostic primer set JMV1 and JMVhapla reported in Wishart et al. (2002). The amplified DNA was then sent to Admera Health Inc. (South Plainfield, NJ, USA) for library preparation using the KAPA HyperPrep PCR-free Kit (Roche Diagnostics Corp., Indianapolis, IN, USA) and sequencing on an Illumina NovaSeq machine. We targeted 30x coverage (∼1.56 Gb) for each sample.

### Mapping and quality control

After verifying that all samples passed quality control with fastQC (Andrews, 2010) and trimming Illumina adaptor sequencing with trimmomatic v.0.36 (Bolger et al., 2014), we aligned raw reads against the chromosome-scale assembly *Meloidogye hapla* from Shakya et al. (2025) using the BWA-MEM algorithm implemented in BWA v.0.717 (Li & Durbin, 2009). We used Picard-tools v.1.141 (Anon, 2019) to remove duplicated reads.

Whole genome amplification relies on large sets of random hexamer primers, which can potentially result in uneven coverage (O’Grady et al., 2022). We estimated coverage breadth, average coverage depth, and coverage evenness for each sample (Oexle, 2016) (Supplementary Table S15). We also computed pairwise correlation coefficients for read counts in 1-kb bins between all individuals to ensure that the expected uneven read distribution resulting from WGA was consistent across samples (Supplementary Figure S7).

### Variant calling and filtering

After removing two individuals with poor alignment to the reference, we were left with 76 individuals for downstream analysis (Supplementary Table S15). We followed the GATK v4.1.7.0 best practices for germline variant calling, which involves using GATK’s HaplotypeCaller and genotypeGCVF algorithms (Supplementary Methods). We further filtered our variant set by removing indels, retaining only biallelic sites, removing variants in low complexity regions, removing variants with less more than 80% missingness, and removing variants with a minor allele counts of less than 3. After all filtering, we retained a list of 149,188 biallelic SNPs.

### Population structure

We inferred population structure using two complementary methods: a standard PCA and admixture analysis. Both analyses require sets of unlinked SNPs, so we pruned SNPs with pairwise LD values above 0.2 using PLINK v2.0 in sliding windows containing 50 SNPs and advancing 5 SNPs at a time (Chang et al. 2015; Purcell & Chang, n.d.). This resulted in 21,225 SNPs for analysis. PCA was performed using the PCA command in the R package SNPrelate (Zheng et al., 2012). Admixture analysis was performed using fastStructure (Raj et al., 2014), implemented in the wrapper program structure_threader (Pina-Martins et al., 2017). We ran fastStructure using admixture proportions of *K* = 1 to *K* = 15. We used the Evanno method to select the best-fitting admixture proportion *K* (Evanno et al., 2005).

Using the same SNP set, we estimated the proportion of genetic variation attributable to sampling location and host plant with a redundancy analysis implemented in the R package vegan (Oksanen et al., 2025).

### Identification of host-associated SNPs

We used two complementary approaches to identify host-associated SNPs using the program BayPass (Gautier, 2015; Olazcuaga et al., 2020). The first approach leveraged all 61 individuals collected from the three sampling sites (BR, SM, and PT) in Virginia (“global”) and the second used only the 29 individuals collected from our most well-sampled site at PT (“local”), minus the 4 individuals assigned to the alternative cytological type. For both approaches, we performed three contrast analyses (between nematodes collected from each pair of host species) implemented in BayPass. Contrast analyses are a categorical variant of genotype-environment association (GEA) model. Full details of how BayPass was implemented appear in the Supplementary Methods. Briefly, both the “core” and “contrast” models were run using default priors for 25,000 Markov chain Monte Carlo (MCMC) iterations with a 5,000-iteration burn-in. The contrast model was provided with the Ω matrix estimated from the core model so that population stratification could be controlled for during estimation of the *C_2_* statistics. Three independent model runs resulted in highly correlated *C_2_* indicating model convergence.

We calibrated *C_2_ estimates* using a POD (Supplementary Methods; Gautier, 2015). We classified SNPs with *C_2_* estimate greater than the 0.99 percentile of the POD as host-associated.

### Inference of selection on host-associated loci

To infer selection on host-associated SNPs, we quantified Tajima’s *D* in non-overlapping sliding windows of 10 kb using the –TajimaD command in vcftools (Danecek et al., 2011). Ten kb roughly equates to the genome-wide average recombination distance of 143 cM/Mb (Thomas et al., 2012). Because our dataset contains evidence of strong population structure, distant population split times, and limited migration among populations (see the *Results* section), we performed our selection inference on our most well-sampled study site, PT, rather than the combined dataset (Simonsen et al., 1995).

We established thresholds for detecting significant balancing and positive selection using the 0.5% and 99.5% quantiles of a distribution of Tajima’s *D* estimated using simulated genetic data (see the *Demographic history estimation and genetic simulations using fastsimcoal* section). We used Fisher exact tests to assess whether 10-kb genomic windows that contained host-associated SNPs were enriched for significantly positive or negative Tajima’s *D* estimates. We also used a simple linear model to test whether the average Tajima’s *D* estimate of 10-kb genomic windows that contain host-associated SNPs were different from those that do not contain host-associated SNPs. Lastly, to determine if elevated Tajima’ *D* values in genomic windows containing host-associated SNPs resulted from the fact that host-associated SNPs are more likely to be at an intermediate frequency, we compared Tajima’s *D* values of genomic windows matched for the number of SNPs they contain and their mean allele frequency. Specifically, for each genomic window containing a host-associated SNP, we first produced a null distribution of matched genomic windows with a similar number of SNPs and mean allele frequency that did not contain a host-associated SNP. Then, for each host-associated window, we computed the percentage of matched genomic windows with a Tajima’s *D* value lower than the host-associated window. We then used a Wilcoxon signed rank test to determine if those percentages were shifted significantly upwards.

### Ancestral recombination graph estimation

To estimate the age in generations of alleles under balancing selection, we inferred ARGs for the PT population sample on the 16 chromosomes of the *M. hapla* using ARGweaver (Rasmussen et al., 2014). Model convergence failed on chromosome 3 and so was excluded from the analysis. We followed Hubisz & Siepel (2020) to run ARGweaver (Supplementary Methods).

### Sequence and gene ontology enrichment analysis

To test whether host-associated SNPs were enriched in specific sequence features, we performed an enrichment analysis using sequence ontology assignments made with snpEff (Cingolani et al., 2012). We first constructed an snpEff genome data for *M. hapla* with the Shakya et al. (2025) assembly. We then queried our full set of 149,188 filtered SNPs against this database to get predicted effect annotations for each SNP. Next, we used the resulting predicted annotations to perform Fisher exact tests to determine whether host-associated SNPs were enriched in the sequence features presented in Figure 5. Genic regions were defined as all exon and intron positions, upstream regions were defined as all positions 1-kb upstream of the first start codon, and downstream regions were defined as all positions 1-kb downstream of the final stop codon.

To test whether host-associated SNPs are enriched for specific molecular functions, we performed a GO term enrichment analysis using the R package topGO (Alexa & Rahnenfuhrer, 2016). GO terms were assigned to annotated genes using InterProScan (v5.76) on default settings. GO term enrichment was performed using 5,668 genes in the *M. hapla* assembly that had annotated GO terms and contained at least one polymorphism. To test for significant enrichment, we used the “classic” algorithm and performed Fisher exact tests.

### Demographic history estimation and genetic simulations using fastsimcoal

To establish significance thresholds for selection inference using Tajima’s *D*, we simulated genetic data from a best-fitting demographic model in fastsimcoal v.2.7 (Excoffier et al., 2021). Detailed methodology for demographic inference and genetic simulations are reported in the Supplementary Methods. Briefly, we tested four different historical migration scenarios: strict isolation (i.e., no migration among demes), continuous migration among all demes for the entire demographic history, continuous migration before the second deme fission only, and continuous migration after the second deme fission only. We tested these four different scenarios on four different tree topologies: three different bifurcating trees and one multifurcating tree, for a total of 16 models.

To establish significance thresholds for selection inference with Tajima’s *D*, we performed 100 independent genetic simulations according to the best-fitting demographic model using fastsimcoal. We quantified Tajima’s *D* in the resulting simulated genetic data in non-overlapping sliding windows of 10 kb using the –TajimaD command in vcftools. The bottom 0.5% and top 99.5% quantile of the distribution of Tajima’s *D* values were used as the threshold for detecting significant positive and balancing selection, respectively (Supplementary Methods).

## Supplementary Material

qrag014_Supplemental_Files

## Data Availability

Raw sequence data are available in the NCBI Sequence Read Archive (SRA) under BioProject accession PRJNA1437807. All codes used to perform the analyses described in this study are available in the Dryad Digital Repository: https://doi.org/10.5061/dryad.ngf1vhj8k
