# Supplementary material for: Evidence for balancing selection on loci associated with host use in the genome of a generalist plant parasite
Source: Evol Lett. 2026 May 7;10(4):355–70. doi: 10.1093/evlett/qrag014 (PMC13451774; doi:10.1093/evlett/qrag014)
Supplement: Supplementary file 1 [file qrag014_supplemental_files.zip › Revised_Supplementary_Material.docx]

**Supplementary Materials**

**Evidence for balancing selection on loci associated with host use in the genome of a generalist plant parasite**

McCall Calvert^1^, Alison Chen^1^, Adya Gupta^1^, Linda Wu^1^, Corlett Wood^1^

^1^Department of Biology, University of Pennsylvania, Philadelphia, PA, USA

**Supplementary methods**

*Whole genome amplification of whole nematodes*

All of the reagents mentioned here were contained in the Qiagen REPLI-g Single Cell Kit (cat. # 150343). Prior to performing whole genome amplification, Buffer D2 was prepared using 1 part 1 M DTT and 11 parts Buffer DLB. A 1:10 dilution of 1 M DTT in H_2_O sc was also prepared beforehand.

To reduce the chance of inadvertently amplifying bacteria and plant DNA, individual nematodes were washed five times in sterile 1X PBS by placing a row of five 1X PBS droplets on a sterile petri dish and carefully and repeatedly drawing the nematode up and down across the 5 droplets. At the final droplet, individual nematodes were crushed with the end of the pipette tip and were carefully drawn up and down with the pipette many times to homogenize the sample. 3 μL of D2 buffer was added to each homogenized whole nematode sample and mixed carefully by flicking. Samples were then incubated at 65 °C for 10 minutes in a dry bath. 3 μL of Stop Solution was then added to each sample. While samples were incubating, a REPLI-g sc polymerase master mix was prepared using a recipe of 6.5 μL H_2_O sc, 29 μL REPLI-g Reaction Buffer, 2.5 μL diluted DTT, and 2 μL REPLI-g sc DNA Polymerase for each sample. 40 uL of master mix was added to each sample and samples were incubated at 30 °C for three hours. After this incubation, samples were incubated at 65 °C for 3 minutes to inactivate the REPLI-g sc DNA Polymerase. Amplification products were quality checked using nanodrop and gel electrophoresis. DNA was quantified using a PicoGreen assay. Reactions resulted in approximately 100-600 ng/μL of amplified product for each sample (Supplementary Table S16).

*Phylogenetic analysis of de-novo mitochondria genome assemblies*

*M. hapla* occurs in at least two cytological forms [(Eisenback and Triantaphyllou 1991)](https://www.zotero.org/google-docs/?lX3Vj9). One form reproduces via facultative meiotic parthenogenesis (i.e. selfing) and the other by obligate mitotic parthenogenesis (i.e. asexual) and they are therefore reproductively isolated. These forms also differ in their chromosome number and ploidy. To determine whether there could be cytological or other subspecific variation in our dataset, we constructed a phylogenetic tree from de-novo mitochondria assemblies. We used GetOrganelle with default settings to assemble mitochondrial genomes for each individual and used the default GetOrganelle animal mitochondrial database to seed the assemblies [(Jin et al. 2020)](https://www.zotero.org/google-docs/?L6A2xe). We then used Ska2 to construct a split *k-*mer based alignment and to perform SNP calling on this set of mitochondria assemblies [(Harris 2018)](https://www.zotero.org/google-docs/?fJVvio). Using these SNPs, we constructed a neighbor-joining tree using VeryFastTree [(Piñeiro et al. 2020)](https://www.zotero.org/google-docs/?pnQyxy).

*Variant calling and hard filtering*

We followed the GATK v.4.1.7.0 best practices workflow for germline variant calling to call variants in our dataset [(McKenna et al. 2010)](https://www.zotero.org/google-docs/?7DqNuK). First we used Picard-tools v.1.141 to generate a reference sequence dictionary required by GATK [(Picard Toolkit 2019)](https://www.zotero.org/google-docs/?wUyCQ4). Then, we used GATK’s HaplotypeCaller function to call SNPs and Indels. HaplotypeCaller is computationally intensive, so we employed an array job whereby an independent instance of HaplotypeCaller was run on each sample. Individual sample genotypes were then combined using GATK’s combineGVCF command. Variants were then called on this combined genotype file using GATK’s genotypeGCVF command. We filtered variants using the standard hard filtering thresholds recommended by the GATK v.4.1.7.0 best practices workflow. Specifically, we filtered SNPs with a quality depth (DP) of less than 2, a mapping quality (MQ) of less than 40, fisher strand (FS) of greater than 60, a MQ rank sum of less than -12.5, and read position rank sum of less than -8.

*Additional BayPass details*

BayPass requires a table of allele counts for each SNP and population in the dataset, which we generated using the -freq counts command in PLINK 2.0. Because we were interested in identifying SNPs associated with host adaptation, we calculated allele counts for nematodes collected from each host plant in each geographic location for a total of 9 populations. We first ran the “core” BayPass model with just the population allele frequency counts to estimate the scaled variance-covariance matrix 𝛀 of population allele frequencies. We ran the core model with default priors, 25,000 MCMC iterations with a burn-in of 5,000 iterations. We ran the core mode 5 times, each with a different seed for the random number generator, to assess model convergence. We found nearly perfect correlations for the posterior means of the elements of 𝛀 matrices across model runs (pearson’s *r* > 0.999 for all pairwise comparisons. Thus, we used one randomly chosen 𝛀 matrix for input in downstream models (Supplementary Figure S8). Next, we ran the “contrast” model in BayPass, which, for each SNP, estimates the mean squared difference of the sum of standardized allele frequencies, called the *C_2_* statistic, of two groups of individuals or populations.

*BayPass POD generation*

The *p*-values of *C_2_* estimates were misbehaved (Supplementary figure S9), potentially owing to the small sample and small number of SNPs used in our study [(Gautier 2015; Olazcuaga et al. 2020)](https://www.zotero.org/google-docs/?tbBR8M). To generate a POD, we simulated population allele frequency counts using the empirical 𝛀 matrix estimated from the core model. We fed the POD through the ‘core’ BayPass model and found that the posterior estimate of the POD 𝛀 matrix was close to the empirical 𝛀 matrix (Forstner and Moonen Distance = 1.259). The similarity of POD and empirical 𝛀 matrices indicates that the POD is faithfully mimicking the real dataset. Next, we performed the contrast model for each pairwise comparison of our host-associated groups of nematodes using the simulated allele counts contained in the POD. This produced what is effectively a null distribution of *C_2_* statistics for each of our three host-associated nematode group comparisons that takes into account the shared ancestry of the populations under study.

*BayPass permutation test*

As an alternative approach to identifying host-associated SNPs, we performed a permutation test with BayPass. This involved shuffling sample labels, with replacement, on the SNP matrix. Label shuffling occurred within sampling locations to preserve BayPass’s ability to control for population structure. Tables of allele counts were generated after each permutation and fed into BayPass as described above. This resulted in a null distribution of permuted *C_2_* for each SNP. If the empirical *C_2_* of a given SNP fell in the 95% tail of the empirical cumulative distribution function, it was classed as significantly host-associated. These tests were one-tailed because *C_2_* statistics are standardized such that a large value is representative of a large allele frequency difference.

A BayPass permutation test performed using all 61 samples from Virginia resulted in a large number of significant host-associated SNPs and effectively a very low significance threshold (Supplementary Figure S3). Out of concern that these permutation tests were inflating type I error, we chose to stick with the more conservative POD thresholding method for classifying host-associated SNPs.

*Ancestral recombination graph inference*

ARGs are a complete record of all recombination and coalescence events since divergence among sequences in a population sample [(Rasmussen et al. 2014)](https://www.zotero.org/google-docs/?cd0gcT). As such, ARGs represent a complete genealogy of every genomic position under study. We used ARGs to derive estimates of the time to the most recent common ancestor (TMRCA) of 10 bp compressed genomic blocks. If a given locus is under balancing selection, then its TMRCA should usually be longer than a locus under weak selective constraints (i.e. neutral).

To estimate ARGs, we first generated mask files for each individual in our sample to mask missing genotype calls. We then used the command arg-sample to infer ARGs using a VCF file containing all individuals from PT and all 149,188 SNPs. Separate instances of arg-sample were executed on single chromosomes at a time. We executed arg-sample with the following parameters: a VCF minimum quality score of 30 (--vcf-min-qual 30), masked any windows of 5 bp that had 2 or more SNPs (--mask-cluster 2,5), weight genotypes by their phred-likelihoods (--use-genotype-probs), a genome-wide mutation rate of 2.0 x 10^-8^ (-m 2.0e-8), a genome-wide recombination rate of 1.4x10^-6^ (-r 1.4e-6), a maximum time point in the model in units of generations of 100 x 10^5^ (--maxtime 100e5), set the delta value for choosing log times as 0.01 (--delta 0.01), and set the site compression at 10 bp (-c 10). All other parameters were left on their default settings. We ran 5000 iterations for each model (--n 5000) with the default thinning interval of 10. Models failed to converge on chromosome 3 and so it was removed from the analysis. We used the command arg-summarize to derive mean and standard deviation calculation of TMRCA estimates across MCMC iterations. We used a burnin of between 3000 iterations.

π_N_/π_S_ *Estimation*

To compute π_N_/π_S_, we followed the method outlined in [Hughes and Nei (1988)](https://www.zotero.org/google-docs/?TidMIo). Briefly, we first generated consensus sequences in fasta format using bcftools for all 11,228 genes in the *M. hapla* assembly for all 76 individuals in our dataset that had more than 30% of reads mapping to the *M. hapla* assembly. We then concatenated consensus sequences for all individuals to generate individual multiple alignment FASTAs (MAF) for each individual gene. For each gene, we computed *d_N_* and *d_S_* for all pairwise combinations of sequences using the method of [Nei and Gojobori (1986)](https://www.zotero.org/google-docs/?UKcVbf) implemented in the BioPython [(Cock et al. 2009)](https://www.zotero.org/google-docs/?bBdFFE) function calculate_dn_ds. Next, we calculated the mean *d_N_* and mean *d_S_* of all pairwise sequence combinations to estimate π_N_ and π_S_, respectively. This allowed us to generate π_N_/π_S_ estimates for all genes.

*Demographic history estimation and genetic simulations using fastsimcoal*

We performed our demographic inference in fastsimcoal v.2.7 [(Excoffier et al. 2021)](https://www.zotero.org/google-docs/?nZH5AA). Prior to analysis with fastsimcoal, we removed SNPs located in genic regions using bedtools [(Quinlan and Hall 2010)](https://www.zotero.org/google-docs/?kKqCuu), and pruned SNPs in high LD (using a cut of < 0.2) in PLINK2.0 [(Chang et al. 2015)](https://www.zotero.org/google-docs/?DzmnlY). We then used easySFS [(Gutenkunst et al. 2009)](https://www.zotero.org/google-docs/?VdTkZJ) to generate a joint three-dimensional minor allele SFS (i.e. a folded SFS) to use as input into fastsimcoal. We used easySFS’s preview function to select the optimal projection.

As mentioned in the main text, We tested four different historical migration scenarios: strict isolation (SI, i.e. no migration among demes), continuous migration among all demes for the entire demographic history, continuous migration before the second deme fission only, and continuous migration after the second deme fission only. We tested these four different scenarios on four different tree topologies: three different bifurcating trees and one multifurcating tree, for a total of 16 models. Models also included the average inbreeding coefficient (Fis) of each population; 0.479 for BR, 0.507 for SM, and 0.19 for PT. For each demographic model, we fit parameters to the observed joint three dimension minor allele SFS using 200,000 coalescent simulations and 50 ECM parameter optimization cycles. 100 independent fits were performed for each model to randomize starting parameter values to increase the chance that we found the global optimum for the combination of parameter values in each of our demographic models.

To select the best fitting demographic model, we first identified the best run of each of the 16 demographic models based on which had the highest likelihood. Then, for each of the 16 demographic models, we performed 100 model runs using the parameters from the initial run with the highest likelihood. These 100 model runs were used to produce likelihood distributions. A continuous migration model with the BR population as the outgroup had the highest likelihood distribution and was selected as the best model. This demographic model also had the best AIC score.

To assess certainty in demographic model parameter estimates we performed a parametric bootstrapping procedure. This involved first simulating 100 three dimensional folded site frequency spectra using the parameters from the best fitting model. Then, for each independent simulation, we estimated demographic parameters using the same estimation and template files used to identify the original best fitting model. We used the distribution of the estimated parameters from the simulated data to produce a 95% bootstrap confidence interval for each parameter.

To produce significance thresholds for significance testing with Tajima’s *D*, we performed 100 independent simulations of genetic data under the best fitting null . For each run, 20 independent chromosomes of 100,000bp in length were simulated. The resulting genotype files were converted to VCFs using custom awk scripts (see dryad repository). Tajima’s *D* was estimated in 10kb non-overlapping windows using the –TajimaD command in vcftools, just as was done for the empirical data. The resulting simulated distribution of Tajima’s *D* values effectively serves as a null expectation of nucleotide variation in the absence of selection. Furthermore, this approach also allows us to reasonably control for the effect of population stratification, population contraction and expansion, migration, and inbreeding on our inference of selection. The 0.5% and 99.5% quantiles of the distribution of the simulated Tajima’s *D* values were used as the significant thresholds for detecting positive and balancing selection, respectively.

**Supplementary Figures and Tables**

**Supplementary Figure S1**: Neighbor-joining tree of SNP variation in sample mitochondria assemblies. Colors represent sampling location. Three distinct clades were revealed corresponding to all samples from PA, the four individuals from PT with nearly complete assignment to the dominant PA population cluster, and the rest of the samples collected across the three sites in Virginia. Thus, PA individuals and four aforementioned PT individuals may belong to a different cytological type or subspecies of *M*. *hapla*.

**Supplementary Figure S2**: Population structure of root-knot nematodes collected at sites in Virginia. (A) Ancestry proportions for each individual root-knot nematode calculated using fastSTRUCTURE with *K* = 4 and colored by the dominant cluster at each sampling location. Each bar represents an individual root-knot nematode and are grouped by the host plant species they were sampled from and sampling location. (B) PCA showing the first and second eigenvectors of genetic variation across root-knot nematodes collected at sites near MLBS. Points represent individuals and are colored by sampling location according to the dominant population clusters identified in the fastSTRUCTURE analysis. Point shapes correspond to the host plant species that individuals were sampled from. In each panel, host plant species are abbreviated; Leu. = *Leucanthemum vulgare*, Med. = *Medicago lupulina*, and Tri. = *Trifolium repens*

**Supplementary figure S3**: Manhattan plots of permutation tests performed with Baypass. The y-axis represents the raw BayPass *C_2_* statistic generated by comparisons of nematodes collected from different host plants at the PT site: (A) Comparison of nematodes collected from *Leucanthemum vulgare* versus *Medicago lupulina* (Leu. - Med.), (B) comparison of nematodes collected from *Leucanthemum vulgare* versus *Trifolium repens* (Leu. - Tri.), (C) comparison of nematodes collected *Medicago lupulina* versus *Trifolium repens* (Tri. - Med.). Points represent the *C_2_* of individual SNPs. Green points are SNPs that were classified as significantly host-associated via the permutation test method.

**Supplementary figure S4**: Manhattan plots of BayPass standardized contrast statistics (*C_2_*) generated by comparisons of nematodes collected from different host plants at the PT site: (A) Comparison of nematodes collected from *Leucanthemum vulgare* versus *Medicago lupulina* (Leu. - Med.), (B) comparison of nematodes collected from *Leucanthemum vulgare* versus *Trifolium repens* (Leu. - Tri.), (C) comparison of nematodes collected *Medicago lupulina* versus *Trifolium repens* (Tri. - Med.). Points represent the *C_2_* of individual SNPs. The red line represents the 0.99% quantile of the pseudo-observed dataset generated for each specific comparison. Points above the red line are classified as significantly host-associated.

**Supplementary Figure S5**: Plot of *C_2_* estimated leveraging samples from all sites (X-axes) versus *C_2_* estimated using samples within the PT site (y-axes) for comparisons between (A) *L. vulgare* and *M. lupulina* nematodes (Leu. - Med.), (B) *T. repens* and *M. lupulina* nematodes (Tri. - Med.), and (C) *L. vulgare* and *T. repens* nematodes (Leu. - Tri.) . SNPs identified as significantly host-associated using samples from all sites are plotted in purple and SNPs identified as significantly host-associated locally in the PT site are plotted in blue. The dashed lines represent the 1% quantile of the PODs generated for outlier detection.

**Supplementary Figures S6**: Tajima’s D values for 10KB windows binned by average allele frequency. (A) Windows containing SNPs classified as host-associated from the entire sample set at Virginia (Global SNPs) do not have higher Tajima’s D values relative to windows that do not contain a host associated SNP. A linear modeling controlling for allele frequency bin found no significant difference in average Tajima’s D values between windows that contained host-associated SNPs and those that did not (F_1,2825_ = 0.2599, P = 0.6108). (B) Windows containing SNPs classified as host-associated locally at PT do have significantly higher Tajima’s D values relative to windows that do not contain a host associated. This pattern is particularly pronounced at intermediate allele frequency bins. A linear modeling controlling for allele frequency bin found a significant difference in average Tajima’s D values between windows that contained host-associated SNPs and those that did not (F_1,2825_ = 6.6875, P = 0.0098).

**Supplementary Figure S7**: Correlation matrix (Pearson’s ρ) for pairwise comparisons of sample normalized read coverage in 1kb windows.

**Supplementary Figure S8:** Principal component analysis of singular-value decomposition of the scaled covariance matrix of allele frequencies 𝛀. Each point represents a host-associated group of nematodes.

**Supplementary Figure S9**: Histogram of *p*-values for SNP-specific *C_2_* estimates for each pairwise comparison of host-associated root-knot nematode groups: (A) *L. vulgare*-associated nematodes vs. *M. lupulina*-associated nematodes (Leu. vs. Med), (B) *L. vulgare*-associated nematodes vs. *T. repens*-associated nematodes (Leu. vs. Tri), (C) *T. repens*-associated nematodes vs. *M. lupulina*-associated nematodes (Tri. vs. Med.)

**Supplementary Table S1**: The number of nematodes collected from each host plant species at each sampling location. One nematode was collected per host plant individual

|  | **Host plant species** | | |
| --- | --- | --- | --- |
| **Sampling location** | ***Medicago lupulina*** | ***Trifolium repens*** | ***Leucanthemum vulgare*** |
| BR | 5 | 5 | 5 |
| SM | 4 | 5 | 7 |
| PT | 11 | 12 | 11 |
| PA | 6 | 3 | 4 |

**Supplementary Table S2**: Parameter estimates and parametric 95% bootstrap confidence intervals from best-fitting demographic model inferred with fastsimcoal. Note that despite this being the best fitting demographic model, our parametric bootstrap procedure generated asymmetric confidence intervals that did not always include the maximum likelihood estimate for some parameters.

| **Parameter** | **Estimate** | **95% parametric bootstrap confidence interval** |
| --- | --- | --- |
| *Effective population sizes* |  |  |
| Bolley Road (BR) | 2,005 | 1,927.5 - 27,183.4 |
| Smallville Fire Road (SM) | 132,254 | 9,844.6 - 106,819.5 |
| Pott’s Mountain Trail (PT) | 94,921 | 5,639.4 - 599,480.7 |
| *Population split times (generations)* |  |  |
| PT - SM | 8,210 | 1,172.7 - 9,881.8 |
| BR - PT | 9,791 | 3,513.8 - 9,951.6 |
| *Contemporary migration rates* |  |  |
| BR to SM | 1.1968e^-4^ | 4.9267e^-5^ - 7.1403e^-3^ |
| SM to BR | 1.593e^-5^ | 1.6219e^-5^ - 1.0023e^-3^ |
| BR to PT | 2.048e^-4^ | 9.9003e^-5^ - 6.0135e^-3^ |
| PT to BR | 8.1351e^-6^ | 2.6102e^-6^ - 1.0418e^-4^ |
| SM to PT | 4.0337e^-7^ | 3.4165e^-8^ - 5.4496e^-5^ |
| PT to SM | 3.6653e^-8^ | 2.8804e^-8^ - 3.2207e^-5^ |
| *Ancient migration rates* |  |  |
| BR to PT | 4.2716e^-5^ | 6.5568e^-8^ - 1.5026^-3^ |
| PT to BR | 5.1828^-7^ | 6.714e^-8^ - 3.2207e^-5^ |
| *Population growth rates (negative values indicate positive growth)* |  |  |
| BR | 5.6322e^-5^ | -3.3989e^-4^ - 8.0918e^-5^ |
| SM | -5.5657e^-4^ | -3.4222e^-3^ - -1.0936e^-4^ |
| PT | -4.5166e^-4^ | -3.0666e^-3^ - -9.2156e^-5^ |

| **Supplementary Table S3**: Significantly enriched molecular function GO-terms for global host-associated SNPs | | | | | |
| --- | --- | --- | --- | --- | --- |
| **GO.ID** | **Term** | **Annotated** | **Significant** | **Expected** | **P-value** |
| GO:0015929 | hexosaminidase activity | 3 | 3 | 0.24 | 0.00052 |
| GO:0001653 | peptide receptor activity | 35 | 8 | 2.81 | 0.00553 |
| GO:0004563 | beta-N-acetylhexosaminidase activity | 2 | 2 | 0.16 | 0.00645 |
| GO:0052866 | phosphatidylinositol phosphate phosphatase activity | 11 | 4 | 0.88 | 0.00862 |
| GO:0008331 | high voltage-gated calcium channel activity | 3 | 2 | 0.24 | 0.01832 |
| GO:0016778 | diphosphotransferase activity | 3 | 2 | 0.24 | 0.01832 |
| GO:0034593 | phosphatidylinositol bisphosphate phosphatase activity | 8 | 3 | 0.64 | 0.02130 |
| GO:0031072 | heat shock protein binding | 14 | 4 | 1.13 | 0.02155 |
| GO:0051119 | sugar transmembrane transporter activity | 14 | 4 | 1.13 | 0.02155 |
| GO:1901681 | sulfur compound binding | 14 | 4 | 1.13 | 0.02155 |
| GO:0004383 | guanylate cyclase activity | 15 | 4 | 1.21 | 0.02757 |
| GO:0005262 | calcium channel activity | 15 | 4 | 1.21 | 0.02757 |
| GO:0000062 | fatty-acyl-CoA binding | 4 | 2 | 0.32 | 0.03471 |
| GO:0000992 | RNA polymerase III cis-regulatory region sequence-specific DNA binding | 4 | 2 | 0.32 | 0.03471 |
| GO:0001016 | RNA polymerase III transcription regulatory region sequence-specific DNA binding | 4 | 2 | 0.32 | 0.03471 |
| GO:0042923 | neuropeptide binding | 4 | 2 | 0.32 | 0.03471 |
| GO:0071617 | lysophospholipid acyltransferase activitivity | 4 | 2 | 0.32 | 0.03471 |
| GO:1901567 | fatty acid derivative binding | 4 | 2 | 0.32 | 0.03471 |
| GO:0015145 | monosaccharide transmembrane transporter activity | 10 | 3 | 0.8 | 0.04045 |
| GO:0015149 | hexose transmembrane transporter activity | 10 | 3 | 0.8 | 0.04045 |
| GO:0004016 | adenylate cyclase activity | 17 | 4 | 1.37 | 0.04233 |
| GO:0015144 | carbohydrate transmembrane transporter activity | 17 | 4 | 1.37 | 0.04233 |

| **Supplementary Table S4**: Significantly enriched biological process GO-terms for global host-associated SNPs | | | | | |
| --- | --- | --- | --- | --- | --- |
| **GO.ID** | **Term** | **Annotated** | **Significant** | **Expected** | **P-value** |
| GO:0034308 | primary alcohol metabolic process | 2 | 2 | 0.15 | 0.0056 |
| GO:0050801 | monoatomic ion homeostasis | 64 | 11 | 4.78 | 0.0070 |
| GO:0000723 | telomere maintenance | 24 | 6 | 1.79 | 0.0070 |
| GO:0032200 | telomere organization | 25 | 6 | 1.87 | 0.0087 |
| GO:0009887 | animal organ morphogenesis | 12 | 4 | 0.9 | 0.0093 |
| GO:0009187 | cyclic nucleotide metabolic process | 19 | 5 | 1.42 | 0.0109 |
| GO:0009190 | cyclic nucleotide biosynthetic process | 19 | 5 | 1.42 | 0.0109 |
| GO:0030203 | glycosaminoglycan metabolic process | 7 | 3 | 0.52 | 0.0115 |
| GO:0048878 | chemical homeostasis | 78 | 12 | 5.82 | 0.0120 |
| GO:0042592 | homeostatic process | 88 | 13 | 6.57 | 0.0127 |
| GO:0098739 | import across plasma membrane | 27 | 6 | 2.02 | 0.0128 |
| GO:0055080 | monoatomic cation homeostasis | 62 | 10 | 4.63 | 0.0153 |
| GO:0006549 | isoleucine metabolic process | 3 | 2 | 0.22 | 0.0159 |
| GO:0008089 | anterograde axonal transport | 3 | 2 | 0.22 | 0.0159 |
| GO:0009097 | isoleucine biosynthetic process | 3 | 2 | 0.22 | 0.0159 |
| GO:0098930 | axonal transport | 3 | 2 | 0.22 | 0.0159 |
| GO:0007168 | receptor guanylyl cyclase signaling pathway | 14 | 4 | 1.05 | 0.0168 |
| GO:0051453 | regulation of intracellular pH | 14 | 4 | 1.05 | 0.0168 |
| GO:0044255 | cellular lipid metabolic process | 195 | 23 | 14.56 | 0.0176 |
| GO:0006182 | cGMP biosynthetic process | 15 | 4 | 1.12 | 0.0215 |
| GO:0006885 | regulation of pH | 15 | 4 | 1.12 | 0.0215 |
| GO:0030641 | regulation of cellular pH | 15 | 4 | 1.12 | 0.0215 |
| GO:0046068 | cGMP metabolic process | 15 | 4 | 1.12 | 0.0215 |
| GO:0006490 | oligosaccharide-lipid intermediate biosynthetic process | 9 | 3 | 0.67 | 0.0247 |
| GO:0048488 | synaptic vesicle endocytosis | 9 | 3 | 0.67 | 0.0247 |
| GO:0140238 | presynaptic endocytosis | 9 | 3 | 0.67 | 0.0247 |
| GO:0010970 | transport along microtubule | 23 | 5 | 1.72 | 0.0247 |
| GO:0099003 | vesicle-mediated transport in synapse | 23 | 5 | 1.72 | 0.0247 |
| GO:0099111 | microtubule-based transport | 23 | 5 | 1.72 | 0.0247 |
| GO:0099504 | synaptic vesicle cycle | 23 | 5 | 1.72 | 0.0247 |
| GO:0065008 | regulation of biological quality | 138 | 17 | 10.3 | 0.0265 |
| GO:0030705 | cytoskeleton-dependent intracellular transport | 24 | 5 | 1.79 | 0.0294 |
| GO:0006873 | intracellular monoatomic ion homeostasis | 50 | 8 | 3.73 | 0.0300 |
| GO:0030003 | intracellular monoatomic cation homeostasis | 50 | 8 | 3.73 | 0.0300 |
| GO:0002164 | larval development | 4 | 2 | 0.3 | 0.0301 |
| GO:0008088 | axo-dendritic transport | 4 | 2 | 0.3 | 0.0301 |
| GO:0009082 | branched-chain amino acid biosynthetic p... | 4 | 2 | 0.3 | 0.0301 |
| GO:0009791 | post-embryonic development | 4 | 2 | 0.3 | 0.0301 |
| GO:0006022 | aminoglycan metabolic process | 10 | 3 | 0.75 | 0.0334 |
| GO:0015749 | monosaccharide transmembrane transport | 10 | 3 | 0.75 | 0.0334 |
| GO:0008643 | carbohydrate transport | 17 | 4 | 1.27 | 0.0334 |
| GO:0046390 | ribose phosphate biosynthetic process | 51 | 8 | 3.81 | 0.0334 |
| GO:0043413 | macromolecule glycosylation | 61 | 9 | 4.55 | 0.0354 |
| GO:0006164 | purine nucleotide biosynthetic process | 52 | 8 | 3.88 | 0.0370 |
| GO:0030258 | lipid modification | 34 | 6 | 2.54 | 0.0375 |
| GO:0045055 | regulated exocytosis | 18 | 4 | 1.34 | 0.0405 |
| GO:0052652 | cyclic purine nucleotide metabolic process | 18 | 4 | 1.34 | 0.0405 |
| GO:0070085 | glycosylation | 63 | 9 | 4.7 | 0.0424 |
| GO:0016079 | synaptic vesicle exocytosis | 11 | 3 | 0.82 | 0.0434 |
| GO:0036465 | synaptic vesicle recycling | 11 | 3 | 0.82 | 0.0434 |
| GO:0046856 | phosphatidylinositol dephosphorylation | 11 | 3 | 0.82 | 0.0434 |
| GO:0030001 | metal ion transport | 136 | 16 | 10.16 | 0.0446 |
| GO:0055082 | intracellular chemical homeostasis | 54 | 8 | 4.03 | 0.0450 |
| GO:0072522 | purine-containing compound biosynthetic process | 54 | 8 | 4.03 | 0.0450 |
| GO:0009152 | purine ribonucleotide biosynthetic process | 45 | 7 | 3.36 | 0.0470 |
| GO:0000054 | ribosomal subunit export from nucleus | 5 | 2 | 0.37 | 0.0478 |
| GO:0000055 | ribosomal large subunit export from nucleus | 5 | 2 | 0.37 | 0.0478 |
| GO:0006560 | proline metabolic process | 5 | 2 | 0.37 | 0.0478 |
| GO:0009071 | serine family amino acid catabolic process | 5 | 2 | 0.37 | 0.0478 |
| GO:0030834 | regulation of actin filament depolymerization | 5 | 2 | 0.37 | 0.0478 |
| GO:0030835 | negative regulation of actin filament depolymerization | 5 | 2 | 0.37 | 0.0478 |
| GO:0033750 | ribosome localization | 5 | 2 | 0.37 | 0.0478 |
| GO:0051016 | barbed-end actin filament capping | 5 | 2 | 0.37 | 0.0478 |
| GO:0051452 | intracellular pH reduction | 5 | 2 | 0.37 | 0.0478 |
| GO:0051693 | actin filament capping | 5 | 2 | 0.37 | 0.0478 |
| GO:0061723 | glycophagy | 5 | 2 | 0.37 | 0.0478 |
| GO:0072359 | circulatory system development | 5 | 2 | 0.37 | 0.0478 |
| GO:0006814 | sodium ion transport | 36 | 6 | 2.69 | 0.0479 |

| **Supplementary Table S5**: Significantly enriched cellular component GO-terms for local host-associated SNPs at PT | | | | | |
| --- | --- | --- | --- | --- | --- |
| **GO.ID** | **Term** | **Annotated** | **Significant** | **Expected** | **P-value** |
| GO:0001401 | SAM complex | 1 | 1 | 0.01 | 0.013 |
| GO:0005673 | transcription factor TFIIE complex | 1 | 1 | 0.01 | 0.013 |
| GO:0016507 | mitochondrial fatty acid beta-oxidation multienzyme complex | 1 | 1 | 0.01 | 0.013 |
| GO:0036125 | fatty acid beta-oxidation multienzyme complex | 1 | 1 | 0.01 | 0.013 |
| GO:0005654 | nucleoplasm | 77 | 4 | 0.97 | 0.015 |
| GO:0005742 | mitochondrial outer membrane translocase complex | 2 | 1 | 0.03 | 0.025 |
| GO:0098799 | outer mitochondrial membrane protein complex | 2 | 1 | 0.03 | 0.025 |
| GO:0005576 | extracellular region | 92 | 4 | 1.16 | 0.027 |
| GO:0035770 | ribonucleoprotein granule | 21 | 2 | 0.26 | 0.028 |
| GO:0036464 | cytoplasmic ribonucleoprotein granule | 21 | 2 | 0.26 | 0.028 |
| GO:1990204 | oxidoreductase complex | 22 | 2 | 0.28 | 0.030 |
| GO:0005739 | mitochondrion | 252 | 7 | 3.17 | 0.034 |
| GO:0098798 | mitochondrial protein-containing complex | 59 | 3 | 0.74 | 0.037 |
| GO:0000110 | nucleotide-excision repair factor 1 complex | 3 | 1 | 0.04 | 0.037 |
| GO:0005688 | U6 snRNP | 3 | 1 | 0.04 | 0.037 |
| GO:0005960 | glycine cleavage complex | 3 | 1 | 0.04 | 0.037 |
| GO:0005615 | extracellular space | 60 | 3 | 0.76 | 0.038 |
| GO:0000109 | nucleotide-excision repair complex | 4 | 1 | 0.05 | 0.049 |

| **Supplementary Table S6**: Significantly enriched molecular function GO-terms for local host-associated SNPs at PT | | | | | |
| --- | --- | --- | --- | --- | --- |
| **GO.ID** | **Term** | **Annotated** | **Significant** | **Expected** | **P-value** |
| GO:0008239 | dipeptidyl-peptidase activity | 1 | 1 | 0.01 | 0.013 |
| GO:0008241 | peptidyl-dipeptidase activity | 1 | 1 | 0.01 | 0.013 |
| GO:0016509 | long-chain-3-hydroxyacyl-CoA dehydrogenase activity | 1 | 1 | 0.01 | 0.013 |
| GO:0016672 | oxidoreductase activity | 1 | 1 | 0.01 | 0.013 |
| GO:0031071 | cysteine desulfurase activity | 1 | 1 | 0.01 | 0.013 |
| GO:0047710 | bis(5'-adenosyl)-triphosphatase activity | 1 | 1 | 0.01 | 0.013 |
| GO:0070224 | sulfide:quinone oxidoreductase activity | 1 | 1 | 0.01 | 0.013 |
| GO:0004713 | protein tyrosine kinase activity | 14 | 2 | 0.18 | 0.013 |
| GO:0016811 | hydrolase activity | 16 | 2 | 0.2 | 0.017 |
| GO:0004300 | enoyl-CoA hydratase activity | 2 | 1 | 0.03 | 0.025 |
| GO:0004563 | beta-N-acetylhexosaminidase activity | 2 | 1 | 0.03 | 0.025 |
| GO:0008494 | translation activator activity | 2 | 1 | 0.03 | 0.025 |
| GO:0016835 | carbon-oxygen lyase activity | 20 | 2 | 0.25 | 0.025 |
| GO:0008238 | exopeptidase activity | 21 | 2 | 0.26 | 0.028 |
| GO:0003857 | 3-hydroxyacyl-CoA dehydrogenase activity | 3 | 1 | 0.04 | 0.037 |
| GO:0004817 | cysteine-tRNA ligase activity | 3 | 1 | 0.04 | 0.037 |
| GO:0015929 | hexosaminidase activity | 3 | 1 | 0.04 | 0.037 |
| GO:0034450 | ubiquitin-ubiquitin ligase activity | 3 | 1 | 0.04 | 0.037 |
| GO:0016810 | hydrolase activity | 28 | 2 | 0.35 | 0.048 |
| GO:0000014 | single-stranded DNA endodeoxyribonuclease activity | 4 | 1 | 0.05 | 0.049 |
| GO:0070403 | NAD+ binding | 4 | 1 | 0.05 | 0.049 |

| **Supplementary Table S7**: Significantly enriched biological process GO-terms for local host-associated SNPs at PT | | | | | |
| --- | --- | --- | --- | --- | --- |
| **GO.ID** | **Term** | **Annotated** | **Significant** | **Expected** | **P-value** |
| GO:0019418 | sulfide oxidation | 1 | 1 | 0.01 | 0.015 |
| GO:0070221 | sulfide oxidation, using sulfide:quinone oxidoreductase | 1 | 1 | 0.01 | 0.015 |
| GO:0001573 | ganglioside metabolic process | 2 | 1 | 0.03 | 0.030 |
| GO:0006689 | ganglioside catabolic process | 2 | 1 | 0.03 | 0.030 |
| GO:0009249 | protein lipoylation | 2 | 1 | 0.03 | 0.030 |
| GO:0019377 | glycolipid catabolic process | 2 | 1 | 0.03 | 0.030 |
| GO:0019464 | glycine decarboxylation via glycine cleavage system | 2 | 1 | 0.03 | 0.030 |
| GO:0043489 | RNA stabilization | 2 | 1 | 0.03 | 0.030 |
| GO:0046479 | glycosphingolipid catabolic process | 2 | 1 | 0.03 | 0.030 |
| GO:0046514 | ceramide catabolic process | 2 | 1 | 0.03 | 0.030 |
| GO:0048255 | mRNA stabilization | 2 | 1 | 0.03 | 0.030 |
| GO:1901255 | nucleotide-excision repair involved in interstrand cross-link repair | 2 | 1 | 0.03 | 0.030 |
| GO:1902373 | negative regulation of mRNA catabolic process | 2 | 1 | 0.03 | 0.030 |
| GO:0016071 | mRNA metabolic process | 125 | 5 | 1.87 | 0.037 |
| GO:0006417 | regulation of translation | 21 | 2 | 0.31 | 0.038 |
| GO:0034248 | regulation of amide metabolic process | 22 | 2 | 0.33 | 0.042 |
| GO:0000395 | mRNA 5'-splice site recognition | 3 | 1 | 0.04 | 0.044 |
| GO:0006423 | cysteinyl-tRNA aminoacylation | 3 | 1 | 0.04 | 0.044 |
| GO:0006546 | glycine catabolic process | 3 | 1 | 0.04 | 0.044 |
| GO:0030149 | sphingolipid catabolic process | 3 | 1 | 0.04 | 0.044 |
| GO:0030203 | glycosaminoglycan metabolic process | 3 | 1 | 0.04 | 0.044 |
| GO:0031086 | nuclear-transcribed mRNA catabolic process | 3 | 1 | 0.04 | 0.044 |
| GO:0031087 | deadenylation-independent decapping of nuclear-transcribed mRNA | 3 | 1 | 0.04 | 0.044 |
| GO:0036297 | interstrand cross-link repair | 3 | 1 | 0.04 | 0.044 |
| GO:0046466 | membrane lipid catabolic process | 3 | 1 | 0.04 | 0.044 |
| GO:1902369 | negative regulation of RNA catabolic process | 3 | 1 | 0.04 | 0.044 |
| GO:1903312 | negative regulation of mRNA metabolic process | 3 | 1 | 0.04 | 0.044 |
| GO:0043085 | positive regulation of catalytic activitivty | 23 | 2 | 0.34 | 0.045 |
| GO:0010629 | negative regulation of gene expression | 55 | 3 | 0.82 | 0.048 |

| **Supplementary Table S8**: Significantly enriched cellular component GO-terms for global host-associated SNPs in genomic windows with significantly positive Tajima’s *D* | | | | | |
| --- | --- | --- | --- | --- | --- |
| **GO.ID** | **Term** | **Annotated** | **Significant** | **Expected** | **P-value** |
| GO:0016272 | prefoldin complex | 7 | 2 | 0.06 | 0.0012 |
| GO:0031436 | BRCA1-BARD1 complex | 1 | 1 | 0.01 | 0.0079 |
| GO:0070531 | BRCA1-A complex | 1 | 1 | 0.01 | 0.0079 |
| GO:0071439 | clathrin complex | 2 | 1 | 0.02 | 0.0158 |
| GO:0045334 | clathrin-coated endocytic vesicle | 5 | 1 | 0.04 | 0.0391 |
| GO:0005764 | lysosome | 43 | 2 | 0.34 | 0.0450 |
| GO:0019773 | proteasome core complex, alpha subunit complex | 6 | 1 | 0.05 | 0.0467 |
| GO:0000323 | lytic vacuole | 44 | 2 | 0.35 | 0.0469 |
| GO:0005789 | endoplasmic reticulum membrane | 112 | 3 | 0.89 | 0.0576 |

| **Supplementary Table S9**: Significantly enriched molecular function GO-terms for global host-associated SNPs in genomic windows with significantly positive Tajima’s *D* | | | | | |
| --- | --- | --- | --- | --- | --- |
| **GO.ID** | **Term** | **Annotated** | **Significant** | **Expected** | **P-value** |
| GO:0044183 | protein folding chaperone | 18 | 3 | 0.16 | 0.00053 |
| GO:0031072 | heat shock protein binding | 14 | 2 | 0.13 | 0.00694 |
| GO:0051082 | unfolded protein binding | 46 | 3 | 0.42 | 0.00823 |
| GO:0003896 | DNA primase activity | 1 | 1 | 0.01 | 0.00914 |
| GO:0004046 | aminoacylase activity | 1 | 1 | 0.01 | 0.00914 |
| GO:0015086 | cadmium ion transmembrane transporter activity | 1 | 1 | 0.01 | 0.00914 |
| GO:0042281 | dolichyl pyrophosphate Man9GlcNAc2 alpha-1,3-glucosyltransferase activity | 1 | 1 | 0.01 | 0.00914 |
| GO:0004095 | carnitine O-palmitoyltransferase activity | 2 | 1 | 0.02 | 0.01821 |
| GO:0004563 | beta-N-acetylhexosaminidase activity | 2 | 1 | 0.02 | 0.01821 |
| GO:0004989 | octopamine receptor activity | 2 | 1 | 0.02 | 0.01821 |
| GO:0005381 | iron ion transmembrane transporter activity | 2 | 1 | 0.02 | 0.01821 |
| GO:0005384 | manganese ion transmembrane transporter activity | 2 | 1 | 0.02 | 0.01821 |
| GO:0016416 | O-palmitoyltransferase activity | 2 | 1 | 0.02 | 0.01821 |
| GO:0016779 | nucleotidyltransferase activity | 63 | 3 | 0.58 | 0.01935 |
| GO:0004866 | endopeptidase inhibitor activity | 26 | 2 | 0.24 | 0.02311 |
| GO:0030414 | peptidase inhibitor activity | 27 | 2 | 0.25 | 0.02482 |
| GO:0061135 | endopeptidase regulator activity | 27 | 2 | 0.25 | 0.02482 |
| GO:0004703 | G protein-coupled receptor kinase activity | 3 | 1 | 0.03 | 0.02719 |
| GO:0015245 | fatty acid transmembrane transporter activity | 3 | 1 | 0.03 | 0.02719 |
| GO:0015929 | hexosaminidase activity | 3 | 1 | 0.03 | 0.02719 |
| GO:0016406 | carnitine O-acyltransferase activity | 3 | 1 | 0.03 | 0.02719 |
| GO:0016715 | oxidoreductase activity | 3 | 1 | 0.03 | 0.02719 |
| GO:0032051 | clathrin light chain binding | 3 | 1 | 0.03 | 0.02719 |
| GO:0061134 | peptidase regulator activity | 30 | 2 | 0.27 | 0.03023 |
| GO:0003785 | actin monomer binding | 5 | 1 | 0.05 | 0.04491 |
| GO:0005049 | nuclear export signal receptor activity | 5 | 1 | 0.05 | 0.04491 |
| GO:0005324 | long-chain fatty acid transporter activity | 5 | 1 | 0.05 | 0.04491 |
| GO:0008227 | G protein-coupled amine receptor activity | 5 | 1 | 0.05 | 0.04491 |
| GO:0051879 | Hsp90 protein binding | 5 | 1 | 0.05 | 0.04491 |
| GO:0004857 | enzyme inhibitor activity | 38 | 2 | 0.35 | 0.04666 |

| **Supplementary Table S10**: Significantly enriched biological process GO-terms for global host-associated SNPs in genomic windows with significantly positive Tajima’s *D* | | | | | |
| --- | --- | --- | --- | --- | --- |
| **GO.ID** | **Term** | **Annotated** | **Significant** | **Expected** | **P-value** |
| GO:0006457 | protein folding | 71 | 5 | 0.59 | 0.00025 |
| GO:0051604 | protein maturation | 113 | 5 | 0.94 | 0.00214 |
| GO:0051083 | 'de novo' cotranslational protein folding | 1 | 1 | 0.01 | 0.00830 |
| GO:0085020 | protein K6-linked ubiquitination | 1 | 1 | 0.01 | 0.00830 |
| GO:0000209 | protein polyubiquitination | 22 | 2 | 0.18 | 0.01391 |
| GO:0001573 | ganglioside metabolic process | 2 | 1 | 0.02 | 0.01653 |
| GO:0006689 | ganglioside catabolic process | 2 | 1 | 0.02 | 0.01653 |
| GO:0006828 | manganese ion transport | 2 | 1 | 0.02 | 0.01653 |
| GO:0010591 | regulation of lamellipodium assembly | 2 | 1 | 0.02 | 0.01653 |
| GO:0034755 | iron ion transmembrane transport | 2 | 1 | 0.02 | 0.01653 |
| GO:0042989 | sequestering of actin monomers | 2 | 1 | 0.02 | 0.01653 |
| GO:1902743 | regulation of lamellipodium organization | 2 | 1 | 0.02 | 0.01653 |
| GO:0003007 | heart morphogenesis | 3 | 1 | 0.02 | 0.02469 |
| GO:0006264 | mitochondrial DNA replication | 3 | 1 | 0.02 | 0.02469 |
| GO:0006450 | regulation of translational fidelity | 3 | 1 | 0.02 | 0.02469 |
| GO:0007508 | larval heart development | 3 | 1 | 0.02 | 0.02469 |
| GO:0008089 | anterograde axonal transport | 3 | 1 | 0.02 | 0.02469 |
| GO:0009411 | response to UV | 3 | 1 | 0.02 | 0.02469 |
| GO:0010976 | positive regulation of neuron projection development | 3 | 1 | 0.02 | 0.02469 |
| GO:0030032 | lamellipodium assembly | 3 | 1 | 0.02 | 0.02469 |
| GO:0042276 | error-prone translesion synthesis | 3 | 1 | 0.02 | 0.02469 |
| GO:0044539 | long-chain fatty acid import into cell | 3 | 1 | 0.02 | 0.02469 |
| GO:0048569 | post-embryonic animal organ development | 3 | 1 | 0.02 | 0.02469 |
| GO:0060491 | regulation of cell projection assembly | 3 | 1 | 0.02 | 0.02469 |
| GO:0061343 | cell adhesion involved in heart morphogenesis | 3 | 1 | 0.02 | 0.02469 |
| GO:0097581 | lamellipodium organization | 3 | 1 | 0.02 | 0.02469 |
| GO:0098930 | axonal transport | 3 | 1 | 0.02 | 0.02469 |
| GO:0120032 | regulation of plasma membrane bounded ce... | 3 | 1 | 0.02 | 0.02469 |
| GO:0140354 | lipid import into cell | 3 | 1 | 0.02 | 0.02469 |
| GO:1901564 | organonitrogen compound metabolic process | 1355 | 17 | 11.24 | 0.02967 |
| GO:0000002 | mitochondrial genome maintenance | 4 | 1 | 0.03 | 0.03279 |
| GO:0002164 | larval development | 4 | 1 | 0.03 | 0.03279 |
| GO:0006577 | amino-acid betaine metabolic process | 4 | 1 | 0.03 | 0.03279 |
| GO:0007507 | heart development | 4 | 1 | 0.03 | 0.03279 |
| GO:0008088 | axo-dendritic transport | 4 | 1 | 0.03 | 0.03279 |
| GO:0009314 | response to radiation | 4 | 1 | 0.03 | 0.03279 |
| GO:0009416 | response to light stimulus | 4 | 1 | 0.03 | 0.03279 |
| GO:0009437 | carnitine metabolic process | 4 | 1 | 0.03 | 0.03279 |
| GO:0009791 | post-embryonic development | 4 | 1 | 0.03 | 0.03279 |
| GO:0010975 | regulation of neuron projection development | 4 | 1 | 0.03 | 0.03279 |
| GO:0019377 | glycolipid catabolic process | 4 | 1 | 0.03 | 0.03279 |
| GO:0031346 | positive regulation of cell projection organization | 4 | 1 | 0.03 | 0.03279 |
| GO:0032042 | mitochondrial DNA metabolic process | 4 | 1 | 0.03 | 0.03279 |
| GO:0046479 | glycosphingolipid catabolic process | 4 | 1 | 0.03 | 0.03279 |
| GO:0046514 | ceramide catabolic process | 4 | 1 | 0.03 | 0.03279 |
| GO:0000731 | DNA synthesis involved in DNA repair | 5 | 1 | 0.04 | 0.04082 |
| GO:0001676 | long-chain fatty acid metabolic process | 5 | 1 | 0.04 | 0.04082 |
| GO:0006301 | postreplication repair | 5 | 1 | 0.04 | 0.04082 |
| GO:0019985 | translesion synthesis | 5 | 1 | 0.04 | 0.04082 |
| GO:0030834 | regulation of actin filament depolymerization | 5 | 1 | 0.04 | 0.04082 |
| GO:0030835 | negative regulation of actin filament depolymerization | 5 | 1 | 0.04 | 0.04082 |
| GO:0051016 | barbed-end actin filament capping | 5 | 1 | 0.04 | 0.04082 |
| GO:0051693 | actin filament capping | 5 | 1 | 0.04 | 0.04082 |
| GO:0072359 | circulatory system development | 5 | 1 | 0.04 | 0.04082 |
| GO:0006458 | 'de novo' protein folding | 6 | 1 | 0.05 | 0.04879 |
| GO:0006826 | iron ion transport | 6 | 1 | 0.05 | 0.04879 |
| GO:0008284 | positive regulation of cell population proliferation | 6 | 1 | 0.05 | 0.04879 |
| GO:0030837 | negative regulation of actin filament polymerization | 6 | 1 | 0.05 | 0.04879 |
| GO:0031333 | negative regulation of protein-containing complex assembly | 6 | 1 | 0.05 | 0.04879 |
| GO:0031344 | regulation of cell projection organization | 6 | 1 | 0.05 | 0.04879 |
| GO:0032272 | negative regulation of protein polymerization | 6 | 1 | 0.05 | 0.04879 |
| GO:0120035 | regulation of plasma membrane bounded cell projection organization | 6 | 1 | 0.05 | 0.04879 |
| GO:0007264 | small GTPase mediated signal transduction | 43 | 2 | 0.36 | 0.04882 |

| **Supplementary Table S11**: Significantly enriched cellular component GO-terms for local host-associated SNPs at PT in genomic regions with significantly positive Tajima’s *D* | | | | | |
| --- | --- | --- | --- | --- | --- |
| **GO.ID** | **Term** | **Annotated** | **Significant** | **Expected** | **P-value** |
| GO:0005847 | mRNA cleavage and polyadenylation specificity factor complex | 7 | 1 | 0.01 | 0.013 |
| GO:0005849 | mRNA cleavage factor complex | 10 | 1 | 0.02 | 0.018 |
| GO:0005764 | lysosome | 33 | 1 | 0.06 | 0.060 |
| GO:0000323 | lytic vacuole | 34 | 1 | 0.06 | 0.061 |
| GO:0005773 | vacuole | 51 | 1 | 0.09 | 0.091 |
| GO:0005615 | extracellular space | 60 | 1 | 0.11 | 0.106 |
| GO:0005576 | extracellular region | 92 | 1 | 0.17 | 0.159 |
| GO:0140513 | nuclear protein-containing complex | 254 | 1 | 0.47 | 0.390 |
| GO:0005634 | nucleus | 736 | 2 | 1.36 | 0.415 |

| **Supplementary Table S12**: Significantly enriched molecular function GO-terms for local host-associated SNPs at PT in genomic regions with significantly positive Tajima’s *D* | | | | | |
| --- | --- | --- | --- | --- | --- |
| **GO.ID** | **Term** | **Annotated** | **Significant** | **Expected** | **P-value** |
| GO:0004563 | beta-N-acetylhexosaminidase activity | 2 | 1 | 0 | 0.0023 |
| GO:0015929 | hexosaminidase activity | 3 | 1 | 0 | 0.0034 |
| GO:0034450 | ubiquitin-ubiquitin ligase activity | 3 | 1 | 0 | 0.0034 |
| GO:0004601 | peroxidase activity | 9 | 1 | 0.01 | 0.0103 |
| GO:0016684 | oxidoreductase activity | 9 | 1 | 0.01 | 0.0103 |
| GO:0016209 | antioxidant activity | 12 | 1 | 0.01 | 0.0137 |
| GO:0043130 | ubiquitin binding | 12 | 1 | 0.01 | 0.0137 |
| GO:0032182 | ubiquitin-like protein binding | 13 | 1 | 0.01 | 0.0148 |
| GO:0061630 | ubiquitin protein ligase activity | 26 | 1 | 0.03 | 0.0294 |
| GO:0061659 | ubiquitin-like protein ligase activity | 29 | 1 | 0.03 | 0.0328 |
| GO:0004842 | ubiquitin-protein transferase activity | 37 | 1 | 0.04 | 0.0417 |
| GO:0004553 | hydrolase activity | 38 | 1 | 0.04 | 0.0428 |
| GO:0016798 | hydrolase activity | 41 | 1 | 0.05 | 0.0461 |
| GO:0020037 | heme binding | 42 | 1 | 0.05 | 0.0472 |
| GO:0046906 | tetrapyrrole binding | 42 | 1 | 0.05 | 0.0472 |

| **Supplementary Table S13**: Significantly enriched biological process GO-terms for local host-associated SNPs at PT in genomic regions with significantly positive Tajima’s *D* | | | | | |
| --- | --- | --- | --- | --- | --- |
| **GO.ID** | **Term** | **Annotated** | **Significant** | **Expected** | **P-value** |
| GO:0001573 | ganglioside metabolic process | 2 | 1 | 0 | 0.0027 |
| GO:0006689 | ganglioside catabolic process | 2 | 1 | 0 | 0.0027 |
| GO:0019377 | glycolipid catabolic process | 2 | 1 | 0 | 0.0027 |
| GO:0046479 | glycosphingolipid catabolic process | 2 | 1 | 0 | 0.0027 |
| GO:0046514 | ceramide catabolic process | 2 | 1 | 0 | 0.0027 |
| GO:0030149 | sphingolipid catabolic process | 3 | 1 | 0 | 0.0041 |
| GO:0030203 | glycosaminoglycan metabolic process | 3 | 1 | 0 | 0.0041 |
| GO:0046466 | membrane lipid catabolic process | 3 | 1 | 0 | 0.0041 |
| GO:0090263 | positive regulation of canonical Wnt signaling pathway | 4 | 1 | 0.01 | 0.0054 |
| GO:0006022 | aminoglycan metabolic process | 5 | 1 | 0.01 | 0.0068 |
| GO:0030177 | positive regulation of Wnt signaling pathway | 5 | 1 | 0.01 | 0.0068 |
| GO:0006687 | glycosphingolipid metabolic process | 6 | 1 | 0.01 | 0.0081 |
| GO:0006378 | mRNA polyadenylation | 8 | 1 | 0.01 | 0.0108 |
| GO:0006672 | ceramide metabolic process | 10 | 1 | 0.01 | 0.0135 |
| GO:0006979 | response to oxidative stress | 10 | 1 | 0.01 | 0.0135 |
| GO:0031124 | mRNA 3'-end processing | 13 | 1 | 0.02 | 0.0175 |
| GO:0060828 | regulation of canonical Wnt signaling pathway | 13 | 1 | 0.02 | 0.0175 |
| GO:0000209 | protein polyubiquitination | 14 | 1 | 0.02 | 0.0189 |
| GO:0030111 | regulation of Wnt signaling pathway | 15 | 1 | 0.02 | 0.0202 |
| GO:0006664 | glycolipid metabolic process | 16 | 1 | 0.02 | 0.0216 |
| GO:1903509 | liposaccharide metabolic process | 16 | 1 | 0.02 | 0.0216 |
| GO:1901136 | carbohydrate derivative catabolic process | 18 | 1 | 0.02 | 0.0242 |
| GO:0006665 | sphingolipid metabolic process | 22 | 1 | 0.03 | 0.0296 |
| GO:0060070 | canonical Wnt signaling pathway | 23 | 1 | 0.03 | 0.0309 |
| GO:0009967 | positive regulation of signal transduction | 25 | 1 | 0.03 | 0.0335 |
| GO:0016055 | Wnt signaling pathway | 25 | 1 | 0.03 | 0.0335 |
| GO:0044242 | cellular lipid catabolic process | 25 | 1 | 0.03 | 0.0335 |
| GO:0031123 | RNA 3'-end processing | 26 | 1 | 0.04 | 0.0349 |
| GO:0198738 | cell-cell signaling by wnt | 26 | 1 | 0.04 | 0.0349 |
| GO:0010647 | positive regulation of cell communication | 29 | 1 | 0.04 | 0.0388 |
| GO:0023056 | positive regulation of signaling | 29 | 1 | 0.04 | 0.0388 |
| GO:0048584 | positive regulation of response to stimulus | 29 | 1 | 0.04 | 0.0388 |
| GO:0016042 | lipid catabolic process | 31 | 1 | 0.04 | 0.0415 |
| GO:0006643 | membrane lipid metabolic process | 33 | 1 | 0.04 | 0.0441 |

**Supplementary Table S14 (see separate excel table):** Genes with known or suspected roles as effectors or cell wall degrading enzymes

| **Supplementary Table S15**: Sequencing and mapping details of the 78 samples involved in this study. Coverage breadth = fraction of the reference genome with at least 1x coverage, Average coverage depth = mean number of reads per genomic position, and Eveness score = a metric, on a scale of 0-1, of the uniformity of read distribution across the reference genome. | | | | | | | |
| --- | --- | --- | --- | --- | --- | --- | --- |
| **Sequence ID** | **Site** | **Host** | **Num. of reads** | **Mapped**  **Reads (%)** | **Coverage breadth (%)** | **Average coverage depth** | **Evenness score** |
| 23048D-02-01 | BR | L | 13,663,521 | 97.33 | 91.5 | 32.46 | 0.72 |
| 23048D-02-02 | BR | L | 16,350,234 | 96.02 | 91.91 | 38.27 | 0.74 |
| 23048D-02-03 | BR | L | 14,319,049 | 96.79 | 92.72 | 33.96 | 0.74 |
| 23048D-02-04 | BR | L | 11,758,238 | 89.88 | 90.92 | 25.86 | 0.66 |
| 23048D-02-05 | BR | L | 11,390,127 | 95 | 91.87 | 26.32 | 0.74 |
| 23048D-02-06 | BR | M | 13,623,744 | 96.6 | 91.12 | 32.07 | 0.7 |
| 23048D-02-07 | BR | M | 10,720,317 | 55.43 | 83.71 | 12.12 | 0.53 |
| 23048D-02-08 | BR | M | 13,432,288 | 90.42 | 90.3 | 29.08 | 0.66 |
| 23048D-02-09 | BR | M | 13,361,267 | 87.71 | 89.84 | 27.99 | 0.62 |
| 23048D-02-10 | BR | M | 14,128,127 | 78.24 | 90.92 | 25.53 | 0.65 |
| 23048D-02-11 | BR | T | 13,505,299 | 91.78 | 90.24 | 30.09 | 0.63 |
| 23048D-02-12 | BR | T | 13,304,241 | 97.08 | 90.93 | 31.45 | 0.69 |
| 23048D-02-13 | BR | T | 12,314,689 | 97.01 | 90.98 | 29.1 | 0.7 |
| 23048D-02-14 | BR | T | 11,527,129 | 84.56 | 90.55 | 22.94 | 0.68 |
| 23048D-02-15 | BR | T | 22,575,608 | 71.52 | 90.08 | 36.32 | 0.58 |
| 23048D-02-16 | PA | L | 17,426,534 | 88.2 | 92.52 | 36.15 | 0.64 |
| 23048D-02-17 | PA | L | 23,585,713 | 74.92 | 94.08 | 42.23 | 0.73 |
| 23048D-02-18 | PA | L | 21,801,256 | 92.39 | 92.44 | 47.68 | 0.67 |
| 23048D-02-19 | PA | L | 14,548,434 | 2.33 | 5.02 | 0.15 | #VALUE! |
| 23048D-02-20 | PA | M | 22,871,811 | 1.42 | 3.69 | 0.16 | #VALUE! |
| 23048D-02-21 | PA | M | 12,351,445 | 88.27 | 91.47 | 25.59 | 0.65 |
| 23048D-02-22 | PA | M | 17,722,664 | 62.36 | 92.21 | 26.11 | 0.68 |
| 23048D-02-23 | PA | M | 15,056,117 | 70.47 | 92.35 | 25.24 | 0.68 |
| 23048D-02-24 | PA | M | 13,176,071 | 81.5 | 87.84 | 25.43 | 0.53 |
| 23048D-02-25 | PA | M | 26,681,300 | 82.37 | 93.11 | 50.93 | 0.68 |
| 23048D-02-26 | PA | T | 13,599,971 | 48.64 | 71.8 | 15.74 | 0.34 |
| 23048D-02-27 | PA | T | 24,782,270 | 76.61 | 91.9 | 43.66 | 0.59 |
| 23048D-02-28 | PA | T | 23,132,512 | 83.66 | 92.79 | 46.26 | 0.65 |
| 23048D-02-29 | SM | L | 11,018,880 | 93.09 | 91.77 | 24.81 | 0.7 |
| 23048D-02-30 | SM | L | 20,114,691 | 89.48 | 91.19 | 43.1 | 0.66 |
| 23048D-02-31 | SM | L | 13,282,031 | 90.54 | 91 | 28.82 | 0.65 |
| 23048D-02-32 | SM | L | 11,402,498 | 92.32 | 89.64 | 25.26 | 0.61 |
| 23048D-02-33 | SM | L | 13,284,372 | 92.26 | 86.96 | 29.6 | 0.47 |
| 23048D-02-34 | SM | L | 14,899,048 | 84.97 | 90.68 | 29.94 | 0.63 |
| 23048D-02-35 | SM | L | 21,448,190 | 95.85 | 92.27 | 50.15 | 0.73 |
| 23048D-02-36 | SM | M | 21,330,934 | 95.1 | 85.84 | 49.02 | 0.46 |
| 23048D-02-37 | SM | M | 18,719,953 | 85.38 | 92.12 | 38.53 | 0.7 |
| 23048D-02-38 | SM | M | 12,955,913 | 96.3 | 87.01 | 30.18 | 0.46 |
| 23048D-02-39 | SM | M | 13,469,291 | 28.54 | 81.09 | 9.33 | 0.53 |
| 23048D-02-40 | SM | T | 13,566,154 | 96.87 | 88.99 | 31.91 | 0.57 |
| 23048D-02-41 | SM | T | 16,993,165 | 68.45 | 90.19 | 26.7 | 0.63 |
| 23048D-02-42 | SM | T | 15,280,357 | 95.12 | 91 | 35.36 | 0.71 |
| 23048D-02-43 | SM | T | 28,360,135 | 94.96 | 91.96 | 65.08 | 0.67 |
| 23048D-02-44 | SM | T | 15,144,595 | 84.26 | 84.46 | 30.39 | 0.43 |
| 23048D-02-45 | PT | L | 17,106,007 | 96.46 | 91.96 | 40.13 | 0.73 |
| 23048D-02-46 | PT | L | 33,079,659 | 88.99 | 91.73 | 69.57 | 0.65 |
| 23048D-02-47 | PT | L | 12,185,015 | 95.68 | 91.67 | 28.34 | 0.72 |
| 23048D-02-48 | PT | L | 12,514,146 | 95.52 | 89.82 | 28.92 | 0.65 |
| 23048D-02-49 | PT | L | 21,079,176 | 91.13 | 91.69 | 46.17 | 0.64 |
| 23048D-02-50 | PT_2 | L | 14,797,890 | 83.03 | 91.15 | 28.67 | 0.67 |
| 23048D-02-51 | PT | L | 27,633,173 | 96.62 | 92.58 | 65.16 | 0.75 |
| 23048D-02-52 | PT | L | 16,833,683 | 95.06 | 91.44 | 38.74 | 0.72 |
| 23048D-02-53 | PT | L | 22,730,498 | 94.24 | 92.13 | 51.91 | 0.74 |
| 23048D-02-54 | PT | L | 15,074,749 | 60.47 | 91.21 | 22.25 | 0.7 |
| 23048D-02-55 | PT | L | 12,575,761 | 97.2 | 90.06 | 29.84 | 0.68 |
| 23048D-02-56 | PT | M | 13,827,424 | 95.64 | 89.89 | 32.06 | 0.66 |
| 23048D-02-57 | PT | M | 14,210,568 | 90.14 | 86.32 | 31.1 | 0.51 |
| 23048D-02-58 | PT | M | 22,403,862 | 96.33 | 90.34 | 51.8 | 0.64 |
| 23048D-02-59 | PT | M | 20,372,906 | 95.98 | 90.49 | 47.18 | 0.62 |
| 23048D-02-60 | PT | M | 25,580,131 | 87.76 | 91.07 | 53.55 | 0.67 |
| 23048D-02-61 | PT | M | 11,989,156 | 86.47 | 89.31 | 24.57 | 0.64 |
| 23048D-02-62 | PT | M | 11,538,707 | 91.78 | 89.83 | 25.41 | 0.67 |
| 23048D-02-63 | PT | M | 19,923,874 | 91.15 | 91.08 | 43.18 | 0.68 |
| 23048D-02-64 | PT_2 | M | 8,420,261 | 90.77 | 89.2 | 17.98 | 0.62 |
| 23048D-02-65 | PT_2 | M | 24,079,197 | 93.97 | 91.83 | 53.56 | 0.58 |
| 23048D-02-66 | PT | M | 13,579,472 | 95.46 | 89.21 | 31.21 | 0.6 |
| 23048D-02-67 | PT | T | 12,863,088 | 96.23 | 90.54 | 30.03 | 0.67 |
| 23048D-02-68 | PT | T | 14,311,943 | 90.68 | 89.9 | 31.03 | 0.62 |
| 23048D-02-69 | PT | T | 20,617,338 | 86.15 | 90.72 | 42.26 | 0.65 |
| 23048D-02-70 | PT | T | 21,172,009 | 96.36 | 90.29 | 49.4 | 0.6 |
| 23048D-02-71 | PT | T | 11,835,133 | 92.36 | 87.03 | 26.39 | 0.53 |
| 23048D-02-72 | PT | T | 12,741,276 | 88.56 | 88.39 | 26.94 | 0.56 |
| 23048D-02-73 | PT | T | 22,180,825 | 97.63 | 90.03 | 52.62 | 0.58 |
| 23048D-02-74 | PT | T | 11,750,336 | 96.33 | 88.69 | 27.39 | 0.6 |
| 23048D-02-75 | PT_2 | T | 21,239,997 | 95.36 | 91.1 | 48.19 | 0.59 |
| 23048D-02-76 | PT | T | 12,232,580 | 94.72 | 88.68 | 28.1 | 0.59 |
| 23048D-02-77 | PT | T | 9,112,758 | 96.39 | 90.67 | 21.34 | 0.73 |
| 23048D-02-78 | PT | T | 15,600,399 | 73.47 | 89.32 | 25.63 | 0.61 |

| **Supplementary Table S16**: Whole genome amplification reaction final DNA concentrations for each sample | | | | | |
| --- | --- | --- | --- | --- | --- |
|  | Sequence ID | Site | Host | REPLI-g amplified product (ng/μl) |  |
|  | 23048D-02-01 | BR | L | 298.00 |  |
|  | 23048D-02-02 | BR | L | 465.45 |  |
|  | 23048D-02-03 | BR | L | 289.98 |  |
|  | 23048D-02-04 | BR | L | 220.59 |  |
|  | 23048D-02-05 | BR | L | 355.82 |  |
|  | 23048D-02-06 | BR | M | 512.61 |  |
|  | 23048D-02-07 | BR | M | 758.56 |  |
|  | 23048D-02-08 | BR | M | 197.07 |  |
|  | 23048D-02-09 | BR | M | 153.96 |  |
|  | 23048D-02-10 | BR | M | 250.97 |  |
|  | 23048D-02-11 | BR | T | 517.51 |  |
|  | 23048D-02-12 | BR | T | 211.77 |  |
|  | 23048D-02-13 | BR | T | 718.39 |  |
|  | 23048D-02-14 | BR | T | 654.69 |  |
|  | 23048D-02-15 | BR | T | 243.13 |  |
|  | 23048D-02-16 | PA | L | 204.91 |  |
|  | 23048D-02-17 | PA | L | 187.27 |  |
|  | 23048D-02-18 | PA | L | 90.26 |  |
|  | 23048D-02-19 | PA | L | 217.65 |  |
|  | 23048D-02-20 | PA | M | 409.71 |  |
|  | 23048D-02-21 | PA | M | 228.43 |  |
|  | 23048D-02-22 | PA | M | 137.30 |  |
|  | 23048D-02-23 | PA | M | 140.24 |  |
|  | 23048D-02-24 | PA | M | 219.61 |  |
|  | 23048D-02-25 | PA | M | 156.90 |  |
|  | 23048D-02-26 | PA | T | 172.58 |  |
|  | 23048D-02-27 | PA | T | 294.09 |  |
|  | 23048D-02-28 | PA | T | 217.49 |  |
|  | 23048D-02-29 | SM | L | 213.73 |  |
|  | 23048D-02-30 | SM | L | 253.91 |  |
|  | 23048D-02-31 | SM | L | 571.40 |  |
|  | 23048D-02-32 | SM | L | 272.53 |  |
|  | 23048D-02-33 | SM | L | 108.88 |  |
|  | 23048D-02-34 | SM | L | 295.07 |  |
|  | 23048D-02-35 | SM | L | 282.33 |  |
|  | 23048D-02-36 | SM | M | 236.27 |  |
|  | 23048D-02-37 | SM | M | 296.05 |  |
|  | 23048D-02-38 | SM | M | 212.75 |  |
|  | 23048D-02-39 | SM | M | 401.88 |  |
|  | 23048D-02-40 | SM | T | 267.63 |  |
|  | 23048D-02-41 | SM | T | 210.79 |  |
|  | 23048D-02-42 | SM | T | 348.96 |  |
|  | 23048D-02-43 | SM | T | 297.02 |  |
|  | 23048D-02-44 | SM | T | 267.63 |  |
|  | 23048D-02-45 | PT | L | 308.78 |  |
|  | 23048D-02-46 | PT | L | 194.13 |  |
|  | 23048D-02-47 | PT | L | 244.11 |  |
|  | 23048D-02-48 | PT | L | 269.59 |  |
|  | 23048D-02-49 | PT | L | 297.02 |  |
|  | 23048D-02-50 | PT | L | 179.44 |  |
|  | 23048D-02-51 | PT | L | 258.81 |  |
|  | 23048D-02-52 | PT | L | 219.61 |  |
|  | 23048D-02-53 | PT | L | 553.76 |  |
|  | 23048D-02-54 | PT | L | 252.93 |  |
|  | 23048D-02-55 | PT | L | 159.84 |  |
|  | 23048D-02-56 | PT | M | 347.98 |  |
|  | 23048D-02-57 | PT | M | 223.53 |  |
|  | 23048D-02-58 | PT | M | 266.65 |  |
|  | 23048D-02-59 | PT | M | 282.33 |  |
|  | 23048D-02-60 | PT | M | 669.39 |  |
|  | 23048D-02-61 | PT | M | 520.44 |  |
|  | 23048D-02-62 | PT | M | 222.55 |  |
|  | 23048D-02-63 | PT | M | 243.13 |  |
|  | 23048D-02-64 | PT | M | 276.45 |  |
|  | 23048D-02-65 | PT | M | 238.23 |  |
|  | 23048D-02-66 | PT | M | 222.55 |  |
|  | 23048D-02-67 | PT | T | 244.11 |  |
|  | 23048D-02-68 | PT | T | 991.78 |  |
|  | 23048D-02-69 | PT | T | 285.27 |  |
|  | 23048D-02-70 | PT | T | 182.38 |  |
|  | 23048D-02-71 | PT | T | 169.64 |  |
|  | 23048D-02-72 | PT | T | 176.50 |  |
|  | 23048D-02-73 | PT | T | 370.52 |  |
|  | 23048D-02-74 | PT | T | 559.64 |  |
|  | 23048D-02-75 | PT | T | 246.07 |  |
|  | 23048D-02-76 | PT | T | 205.89 |  |
|  | 23048D-02-77 | PT | T | 235.29 |  |
|  | 23048D-02-78 | PT | T | 231.37 |  |

**References**

Chang, C. C., C. C. Chow, L. C. Tellier, S. Vattikuti, S. M. Purcell, and J. J. Lee. 2015. Secondgeneration PLINK: rising to the challenge of larger and richer datasets. GigaScience 4:s13742-015-0047–8.

Cock, P. J. A., T. Antao, J. T. Chang, B. A. Chapman, C. J. Cox, A. Dalke, I. Friedberg, T. Hamelryck, F. Kauff, B. Wilczynski, and M. J. L. de Hoon. 2009. Biopython: freely available Python tools for computational molecular biology and bioinformatics. Bioinformatics 25:1422–1423.

Eisenback, J. D., and H. H. Triantaphyllou. 1991. Root-knot nematodes: Meloidogyne species and races. Pp. 191–274 in W. R. Nickle, ed. Manual of Agricultural Nematology. Marcell Dekker, New York.

Excoffier, L., N. Marchi, D. A. Marques, R. Matthey-Doret, A. Gouy, and V. C. Sousa. 2021. fastsimcoal2: demographic inference under complex evolutionary scenarios. Bioinformatics 37:4882–4885.

Gautier, M. 2015. Genome-Wide Scan for Adaptive Divergence and Association with Population-Specific Covariates. Genetics 201:1555–1579.

Gutenkunst, R. N., R. D. Hernandez, S. H. Williamson, and C. D. Bustamante. 2009. Inferring the Joint Demographic History of Multiple Populations from Multidimensional SNP Frequency Data. PLOS Genet. 5:e1000695. Public Library of Science.

Harris, S. R. 2018. SKA: Split Kmer Analysis Toolkit for Bacterial Genomic Epidemiology. bioRxiv.

Hughes, A. L., and M. Nei. 1988. Pattern of nucleotide substitution at major histocompatibility complex class I loci reveals overdominant selection. Nature 335:167–170.

Jin, J.-J., W.-B. Yu, J.-B. Yang, Y. Song, C. W. dePamphilis, T.-S. Yi, and D.-Z. Li. 2020. GetOrganelle: a fast and versatile toolkit for accurate de novo assembly of organelle genomes. Genome Biol. 21:241.

McKenna, A., M. Hanna, E. Banks, A. Sivachenko, K. Cibulskis, A. Kernytsky, K. Garimella, D. Altshuler, S. Gabriel, M. Daly, and M. A. DePristo. 2010. The Genome Analysis Toolkit: A MapReduce framework for analyzing next-generation DNA sequencing data. Genome Res. 20:1297–1303.

Nei, M., and T. Gojobori. 1986. Simple methods for estimating the numbers of synonymous and nonsynonymous nucleotide substitutions. Mol. Biol. Evol. 3:418–426.

Olazcuaga, L., A. Loiseau, H. Parrinello, M. Paris, A. Fraimout, C. Guedot, L. M. Diepenbrock, M. Kenis, J. Zhang, X. Chen, N. Borowiec, B. Facon, H. Vogt, D. K. Price, H. Vogel, B.

Prud’homme, A. Estoup, and M. Gautier. 2020. A Whole-Genome Scan for Association with Invasion Success in the Fruit Fly Drosophila suzukii Using Contrasts of Allele Frequencies Corrected for Population Structure. Mol. Biol. Evol. 37:2369–2385.

Piñeiro, C., J. M. Abuín, and J. C. Pichel. 2020. Very Fast Tree: speeding up the estimation of phylogenies for large alignments through parallelization and vectorization strategies. Bioinformatics 36:4658–4659.

Quinlan, A. R., and I. M. Hall. 2010. BEDTools: a flexible suite of utilities for comparing genomic features. Bioinformatics 26:841–842.

Rasmussen, M. D., M. J. Hubisz, I. Gronau, and A. Siepel. 2014. Genome-Wide Inference of Ancestral Recombination Graphs. PLOS Genet. 10:e1004342. Public Library of Science. 2019. Picard Toolkit. Broad Institute
